# Nonintuitive Surface Self-Assembly of Functionalized Molecules on Ag(111)

**DOI:** 10.1021/acsnano.0c10065

**Published:** 2021-03-17

**Authors:** Andreas Jeindl, Jari Domke, Lukas Hörmann, Falko Sojka, Roman Forker, Torsten Fritz, Oliver T. Hofmann

**Affiliations:** † Institute of Solid State Physics, NAWI Graz, Graz University of Technology, Petersgasse 16, 8010 Graz, Austria; ‡ Institute for Solid State Physics, Friedrich Schiller University Jena, Helmholtzweg 5, 07743 Jena, Germany

**Keywords:** organic/inorganic interface, density functional theory, scanning tunneling microscopy, low energy electron diffraction, structure prediction, design principle, molecular driving forces

## Abstract

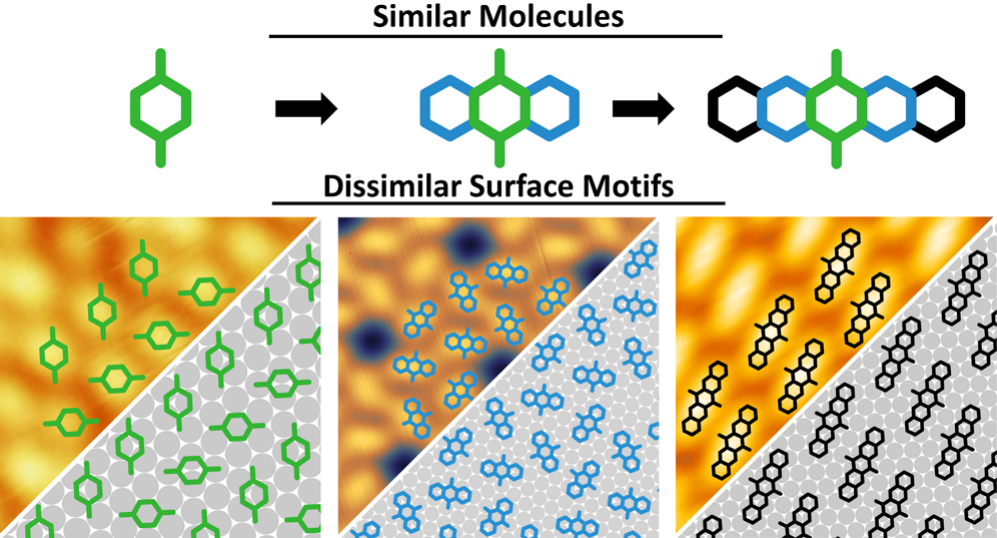

The fabrication of nanomaterials involves self-ordering processes of functional molecules on inorganic surfaces. To obtain specific molecular arrangements, a common strategy is to equip molecules with functional groups. However, focusing on the functional groups alone does not provide a comprehensive picture. Especially at interfaces, processes that govern self-ordering are complex and involve various physical and chemical effects, often leading to unexpected structures, as we showcase here on the example of a homologous series of quinones on Ag(111). Naively, one could expect that such quinones, which all bear the same functionalization, form similar motifs. In salient contrast, our joint theoretical and experimental study shows that profoundly different structures are formed. Using a machine-learning-based structure search algorithm, we find that this is due to a shift of the balance of three antagonizing driving forces: adsorbate–substrate interactions governing adsorption sites, adsorbate–adsorbate interactions favoring close packing, and steric hindrance inhibiting certain otherwise energetically beneficial molecular arrangements. The theoretical structures show excellent agreement with our experimental characterizations of the organic/inorganic interfaces, both for the unit cell sizes and the orientations of the molecules within. The nonintuitive interplay of similarly important interaction mechanisms will continue to be a challenging aspect for the design of functional interfaces. With a detailed examination of all driving forces, we are, however, still able to devise a design principle for self-assembly of functionalized molecules.

Many properties of thin films, such as optical properties^1^ or electrical conductivity,^2^ are determined by the structure that the films assume upon adsorption on a substrate.^3^ To engineer functional interfaces, it is therefore imperative to understand and predict which structures form for a given material combination.^4−8^ At a single molecule level, relevant handles to influence their properties are well-known. A typical example is the conjuction length of organic molecules, which are relevant for organic nanoelectronics.^9−11^ For these, increasing the size of the π-electron backbone or introducing functional groups systematically affects optical properties.^12−15^ At the same time, changing either the backbone or the functional groups will also change the molecule’s crystal polymorphs and its physical properties^16−19^ in nonobvious ways. Particularly for thin films, and even more so for monolayers, the complex interplay between intermolecular and molecule–surface interactions can lead to the formation of packing motifs not observed in the bulk.^3^

A starting point to design complex adsorbate layers with specific properties is to exploit diverse chemical design principles. Previously probed design principles include noncovalent interactions,^20−29^ halogen bonding,^30−33^ dipole–dipole interactions,^34−38^ or steric blocking^39^ and shape complementarity.^23,28,29,32^ When one of these interactions is dominant, an intuitive guess of the resulting motifs can be made. Unfortunately, at interfaces, the interplay with the surface often thwarts this approach. Therefore, a typical approach to design such molecules is to combine these design principles with increasing empirical knowledge from preceding experiments, enabled by molecular-resolution scanning tunneling microscopy (STM). The driving forces leading to self-assembly can then, in hindsight, be analyzed by experimental and theoretical methods. However, even when the driving forces for a specific system are known, a holistic design of particular motifs is typically still prevented by the fact that the knowledge of the driving forces cannot be easily transferred from one case to another. Quite contrarily, even systems that have similar interactions can form disparate structures, as we demonstrate hereafter.

In this work, we examine the predictability and transferability of driving forces for on-surface molecular structures. For this, we use a homologous series of molecules with identical functionalization, namely, the quinones 1,4-benzoquinone (B2O), 9,10-anthraquinone (A2O), and 6,13-pentacenequinone (P2O). In all three molecules, the functional groups provide directed forces for self-assembly via (i) highly attractive intermolecular interactions between the oxygens and hydrogens and (ii) a strong molecule–substrate interaction due to surface-induced aromatic stabilization.^40^ Conversely, the backbone interacts much less site-specifically via van der Waals forces. When depositing up to a single monolayer on Ag(111), we find three entirely different structures (overview in Figure 1; a detailed characterization is given later in this work). While the smallest molecule, B2O, exhibits a simple 2D surface pattern, the larger A2O forms symmetric hexagonal rings with voids between them. The largest molecule of this series, P2O, crystallizes in close-packed molecular rows, as also found by others.^40,41^ Despite the chemical similarity, the backbone size thus decisively determines which motifs form.

**Figure 1:**
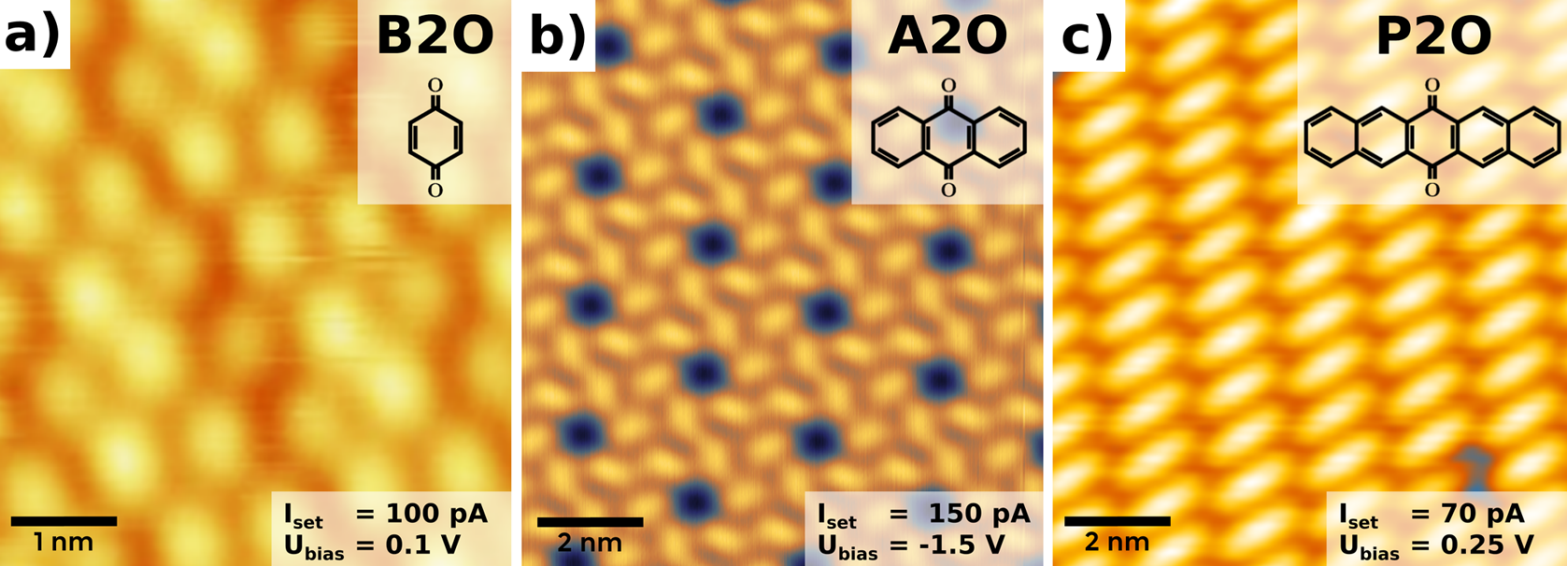
Comparison of experimental constant-current scanning tunneling microscopy (STM) images for molecular monolayers of (a) 1,4-benzoquinone (B2O), (b) 9,10-anthraquinone (A2O), and (c) 6,13-pentacenequinone (P2O) on a Ag(111) surface prepared by physical vapor deposition in ultrahigh vacuum.

We provide systematic insight into how the backbone size affects the driving forces leading to the formation of these motifs. To this end, we predict, based on first-principles, which on-surface motifs the three quinones form on Ag(111), utilizing a combination of density functional theory (DFT) and machine learning (for details, see Methods). First, we focus on the interactions of individual molecules with the Ag(111) surface to unveil trends in the molecule–substrate interaction. We then investigate the intermolecular interactions of close-packed molecular layers on the Ag surface. Mapping the intermolecular energies onto specific molecule parts enables us to identify the main contributors for the motif formation. This procedure allows extraction of general trends for those interactions. We also find that the aspect ratio of the molecules plays a large (and hitherto probably undervalued) role, determining how many favorable interactions with neighboring adsorbates a single molecule can obtain. Finally, a detailed examination of all driving forces for the experimentally observed structures allows us to devise a design principle for the applicability of functional groups to tailor molecular self-assembly on surfaces.

## Results and Discussion

### Interaction of Individual Molecules with Ag(111)

Generally, individual quinone-functionalized molecules interact with the Ag(111) surface by site-specific interactions between the oxygens and the metal, as well as via much less site-specific van der Waals interactions between backbone and substrate. One could thus expect that in the absence of intermolecular interactions all three different quinones prefer the same or similar adsorption geometries on the metal (defined by the adsorption site on the metal substrate and azimuthal rotation of the molecule). To test whether this is indeed the case, we first performed a prescreening of a potential energy surface (PES) with reduced dimensionality, followed by full geometry optimizations (see details in Methods).

**Figure 2:**
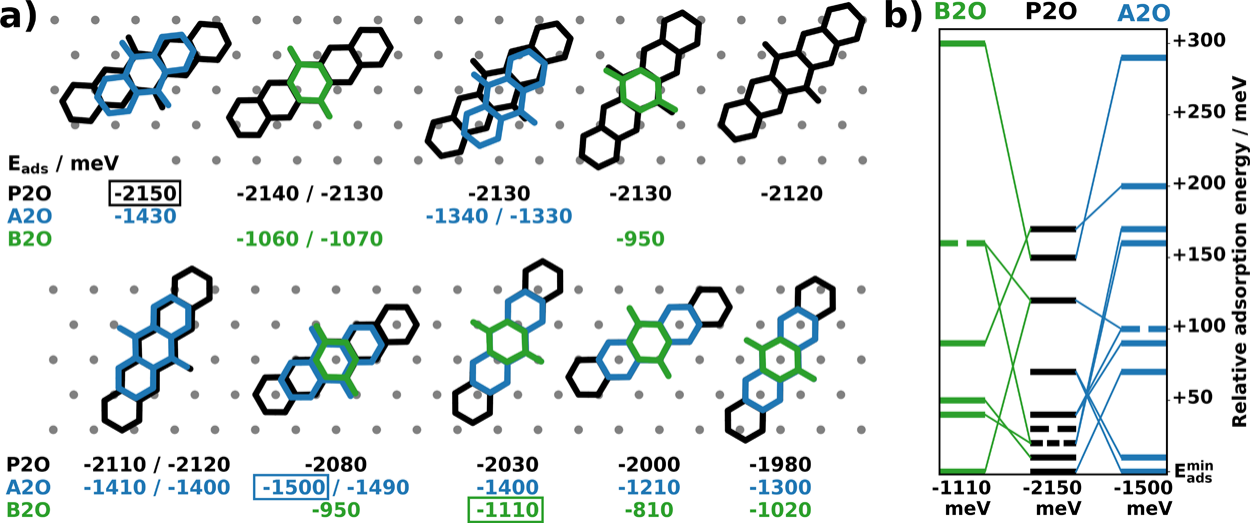
(a) Visualization of all calculated symmetry-inequivalent adsorption geometries for B2O, A2O, and P2O molecules with their corresponding adsorption energies (negative values of *E*_ads_ denote energy gain upon adsorption). Substrate surface atom positions are indicated with gray dots. Boxes mark the energetically best adsorption geometries. All energy values are given in meV. Two energies for a single visualized geometry mark hcp- and fcc-hollow sites, respectively (i.e., there are two adsorption geometries that only differ due to stacking of the substrate layers; the left energy corresponds to the geometry shown here (hcp hollow site). (b) Spread of all adsorption geometry energies for all three molecules relative to the energy of the respective best geometry (E_^ads^_^_min_^. Adsorption energies with multiple energetically equivalent geometries are indicated with dashed lines; the number of dashes is equal to the number of geometries. The colored connecting lines indicate the energetic reordering compared to the P2O geometries.

To compare the three molecules, we focus on three important aspects, which are visualized in Figure 2: (i) the adsorption geometries, (ii) the adsorption energies, and (iii) their energy distribution (i.e., how many adsorption geometries exist within a certain energy range). Despite different backbone lengths, the adsorption geometries are rather similar, while the number of different adsorption geometries increases with backbone size. As a consequence, all adsorption geometries of B2O and A2O feature a corresponding adsorption geometry of P2O, where the oxygens are approximately in the same positions. Figure 2a illustrates this point, showing the P2O geometries sorted by their adsorption energy and overlaying the corresponding B2O and A2O geometries.

Although the adsorption geometries are very similar for the three molecules, their energetic ranking is very different (Figure 2b). This suggests a key-lock-like interaction between the quinones and the surface. Presumably, which geometries are stable is governed (mostly) by the oxygens that bind to specific sites on the Ag(111) surface. Conversely, their energetic ranking depends on the registry of the backbone with the surface. For the most stable geometries, the adsorption energy increases with increasing backbone size (−1.11 eV for B2O, −1.50 eV for A2O, −2.15 eV for P2O), as expected.

As introduced before, the second factor determining which motifs form is the intermolecular interaction. An energetically unfavorable adsorption geometry might still be part of the energetically most favorable motif if it accommodates more attractive intermolecular interactions compensating the loss in adsorption energy.

For our systems, the spread of possible adsorption energies decreases with increasing molecule size from 300 meV for B2O to 170 meV for P2O. Concomitantly, an increasing number of stable adsorption geometries is found energetically close to the most stable geometry, making more adsorption geometries easily accessible for monolayer formation. Especially for P2O, already 8 of its 12 adsorption geometries are found within a range of 50 meV. In general, making the molecule larger by adding benzene rings to the quinone backbone increases the number of adsorption geometries and increases adsorption energies. Simultaneously, the energy difference between different minima decreases, leading to a weaker adsorption-geometry dependence. This is a direct consequence of the inherent mismatch between the acene backbone and the Ag(111) periodicity: the larger the backbone, the more the energetic landscape of the molecule–substrate potential becomes smoothed out.

### Intermolecular Interactions on the Surface

Now that we understand interactions of individual molecules with the surface, we proceed to the intermolecular interactions on the surface. The number of possible arrangements of molecules on the surface, however, is enormous,^42^ which makes an exhaustive mapping of the interactions intractable. This challenge is a common problem for theoretical surface structure search methods. Previous approaches to tackle it include approximating intermolecular interactions with gas phase data^43^ or via modified force fields.^44^ Those methods perform best for low coverages or weak molecule–surface interactions. Here, however, the focus is on close-packed structures with strong molecule–surface interactions, rendering those methods inapplicable.

**Figure 3:**
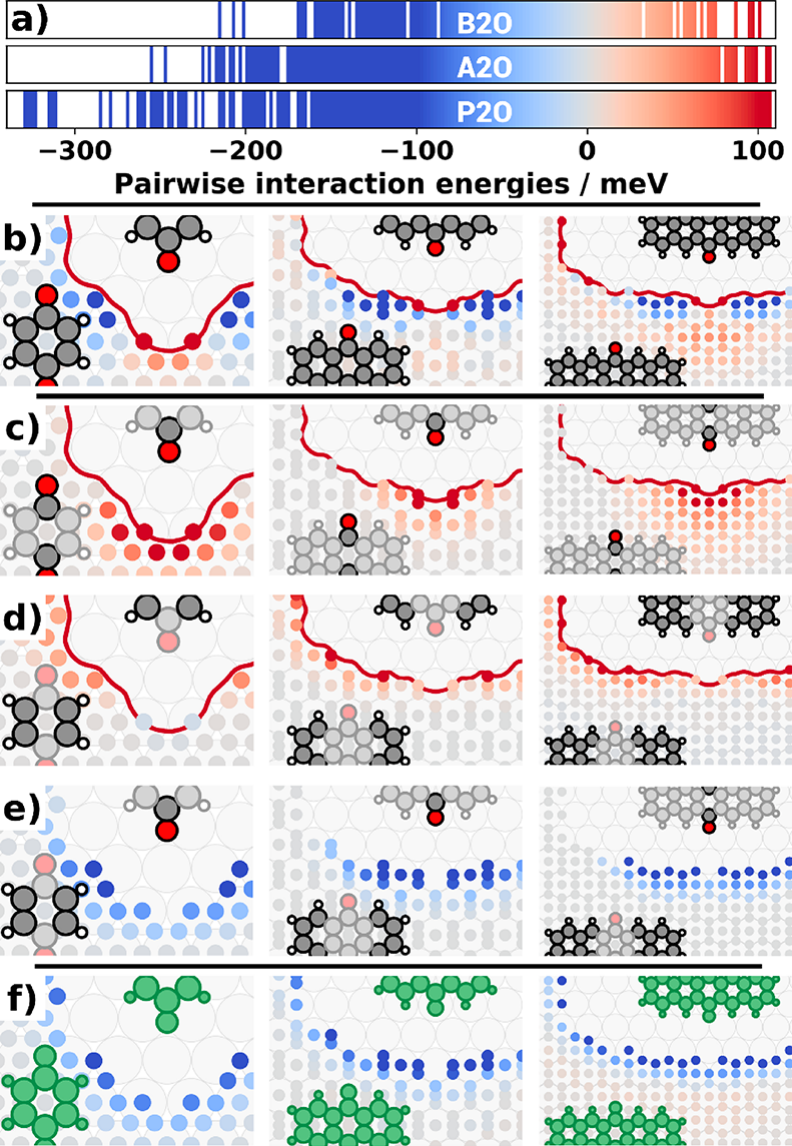
Visualization of pairwise intermolecular energies and their contributions. (a) Distribution of interaction energies for the three molecules under investigation. Colored areas represent interactions present in the given energy window. (b) Total pairwise interaction energies for the three quinones. Each circle represents a possible interaction between the central molecule and another molecule centered at the circle position. The red contour shows the minimal distance before a pair is considered colliding. The circle color (same color scale as in panel a) indicates the corresponding interaction energy. (c–e) Electronic interaction energies between molecules mapped onto different molecule parts: (c) oxygen–oxygen, (d) hydrogen–hydrogen, (e) oxygen–hydrogen; (f) van der Waals interactions between molecules.

For this reason, we used the SAMPLE^45^ approach. It uses the previously mentioned adsorption geometries as constituents to build a large but discrete set of potential motifs with (here) up to six molecules per unit cell and various unit cell sizes (see details in Methods). From the millions of potential motifs, we selected approximately 250 of the most diverse candidates using D-optimality^46^ and calculated their formation energies using DFT. These calculations were then used to infer all relevant molecule–substrate and intermolecular interactions on the surface using the energy model (eq 1) with Bayesian linear regression.
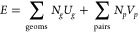
1Here, *U*_*g*_ is the adsorption energy of a molecule with the adsorption geometry *g*, while *V*_*p*_ is the interaction energy between every pair of molecules (called “pairwise interaction” hereafter) in the motif. *N*_*g*_ and *N*_*p*_ denote how often the corresponding interactions appear in each motif. With this method, we can predict the formation energies for all potential motifs with a leave-one-out cross validation error (as explained in the Methods section) of less than 20 meV per adsorbate molecule. Figure 3a gives an overview over the intermolecular interaction energies (*V*_*p*_) for the three systems investigated. The most attractive interactions lead to an energy gain of up to 200 meV for a pair of B2O molecules, 250 meV for A2O, and 300 meV for P2O. Hence, for larger molecules also the intermolecular interactions become stronger. We also estimated the energetic contributions of the interactions between the adsorption-induced dipoles for all tight-packed motifs (see details in Methods). The interaction energies of those dipole sheets are all below 6 meV per molecule, rendering adsorption-induced dipole–dipole interactions a negligible factor for the systems presented in this work.

Besides the total interaction energies within the motifs, the specific form of the energy model also allows us to extract and visualize all pairwise interactions used in the model. Figure 3b shows these interactions spatially resolved for B2O, A2O, and P2O. In these interaction plots, the molecule in the center is kept fixed and the second molecule is moved (at a fixed rotation relative to the substrate) to all different possible adsorption sites around it. Each circle indicates the center of the second molecule with the color of the circle corresponding to the interaction energy of this molecular pair. The discretization of the pairwise interactions stems from the usage of adsorption geometries as building blocks. For the sake of clarity, Figure 3 visualizes only the intermolecular interactions of parallel oriented molecules. The interactions of differently rotated molecules and the overall influence of intermolecular rotation on the interaction energy are given in the Supporting Information (Figures S9 and S10).

The general form of interactions is similar for all three systems and independent of the size of the backbone.

To obtain a deeper insight into the driving forces, we first separated the total interaction energy *E* into contributions from the van der Waals correction, *E*^vdW^ (as given by TS^surf^^47,48^), and the electronic contributions, *E*^elec^ (as given by the PBE^49^ exchange-correlation functional). *E*^vdW^ and *E*^elec^ were then fitted separately using eq 1. As a second step, we mapped the intermolecular interactions of *E*^elec^ onto specific parts by breaking up *V*_*p*_ into a sum of fragments, *V*_*p*_^*f*^:
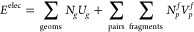
2

Figure 3c–e shows this mapping for the interactions between oxygens (c), between carbon rings (d) and the interactions between oxygens and rings (e). The van der Waals contributions are shown in a separate panel (Figure 3f). In passing, we note that the mapping procedure may, in principle, have multiple stable solutions that yield combined formation energies with comparable accuracy. However, at least for the present systems, it proved to be very robust, providing essentially the same results when started with different initial guesses for the parameters.

The intermolecular oxygen interactions (Figure 3c) are exclusively repulsive, following Coulomb-like behavior due to the partially negatively charged oxygen atoms on both molecules. Interactions between rings (Figure 3d) are dominated by the proximity of hydrogen atoms and are also purely repulsive. The only attractive electronic interactions occur between carbon rings and oxygens (Figure 3e). The second, highly attractive contribution stems from van der Waals interactions between molecules (Figure 3f). This is surprising insofar as the molecules are all lying flat on the surface, which results in a relatively small contact area between adjacent molecules. While those results are qualitatively consistent with expectations, we resolve the interactions based on chemical groups in a quantitative and position-specific way, revealing the general intermolecular interaction characteristics.

### Stable Motifs in Theory and Experiment

Having characterized all relevant interactions on the surface, we can now evaluate which motifs are expected to be observed. For this, we evaluated the energy model (eq 1) for all the millions of candidates and identified the best motifs in terms of formation energy per molecule. The best motifs for all three molecules are presented in Figure 5, and further details of the prediction process are given in section 3 of the Supporting Information. In the following, we focus on the structures predicted to be energetically most favorable and their comparison to the experimental thin films prepared via physical vapor deposition in ultrahigh vacuum. We note that several phases can form upon deposition, depending on the processing conditions and the coverage (see details in Methods). Extensive structural diversity is not uncommon for inorganic/organic interfaces prepared by physical vapor deposition,^50,51^ owing to the potential existence of multiple polymorphs as well as kinetic trapping. Here, we focus on the structures that can be prepared reproducibly and show long-term stability under measurement conditions and for which we are confident that neither surface-adatoms are contained nor surface defects play a major role, that is, the structures where we can reasonably expect that the thermodynamic equilibrium has been reached in the experiment. A detailed description of all other structures found will be provided elsewhere.

**Figure 4:**
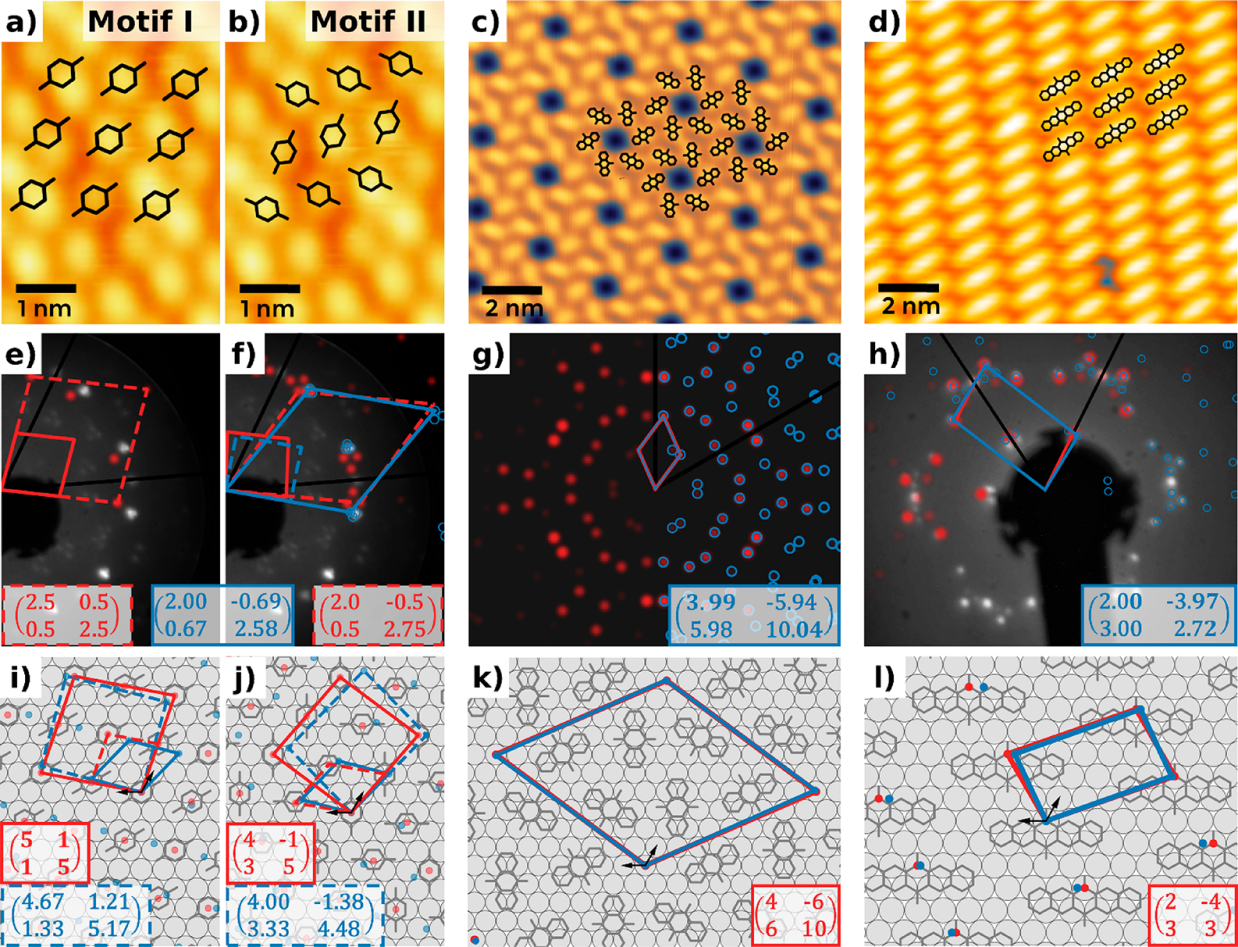
Comparison of theoretical findings (red) with experimental results (blue) and interpretations thereof. (a–d) Comparison of STM experiments (see Figure 1) with theoretically predicted surface polymorphs. (e–h) Comparison of theoretical LEED patterns obtained via kinematic diffraction theory (red) to fits of FFTs from STM images (blue) and LEED images; primary electron energies are 27 eV for B2O and 48 eV for P2O. (i–l) Visualization of the real-space on-surface arrangement. The epitaxy matrices represent the unit cells in the respective substrate basis given by the black arrows. Fit uncertainties for the experimental epitaxy matrices (blue) are below 0.08 for all elements (details in Supporting Information, Table S2). For B2O, two possible theoretical phases are shown. While LEED experiments reveal the primitive adsorbate unit cell, simulations for B2O require 4 molecules in a commensurate supercell. The dashed unit cells in panels e–f and i–j represent transformed cells to obtain comparability (experimental cells replicated; simulated cells reduced).

The qualitative agreement between theory and experiment can already be seen in Figure 4a–d. To obtain a quantitative insight, we compare both the experimental low energy electron diffraction (LEED) images and diffraction patterns obtained via the fast Fourier transform (FFT) of the scanning tunneling microscopy (STM) images (see details in Supporting Information, section 2), with kinematic scattering simulations for the predicted structures, taking into account the tabulated structure factors for all atoms (see Methods). Figure 4e–h compares the experimental diffraction patterns and deduced unit cells for B2O, A2O, and P2O to our best-fitting low-energy predictions.

**Figure 5:**
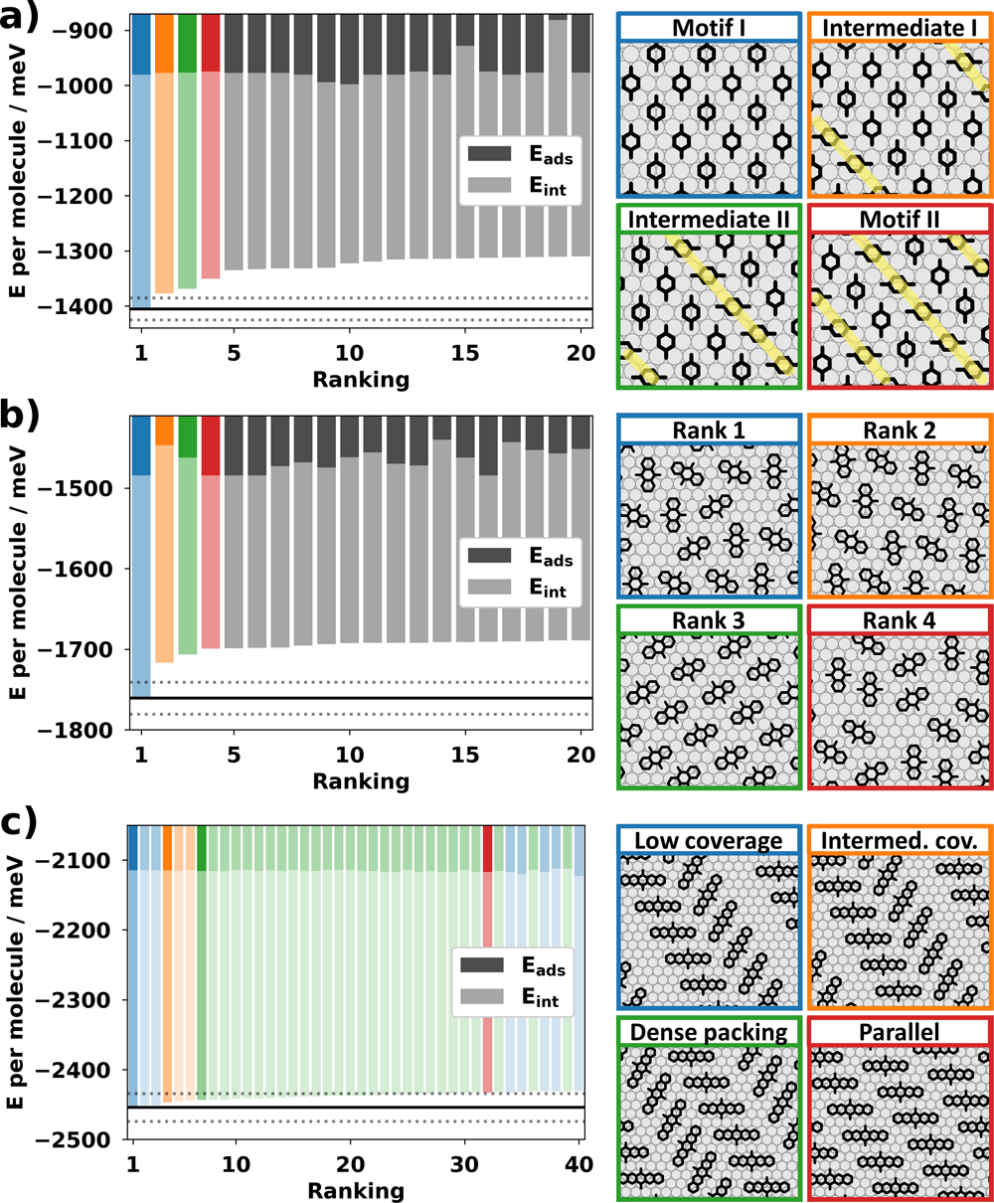
(left) Energies for the energetically most favorable motifs split into molecule–substrate (*E*_ads_) and molecule–molecule (*E*_int_) interactions. The energy uncertainty of 20 meV is visualized with dotted lines relative to the best motif (solid line). Relevant motifs for all three molecules are shown on the right: For B2O (a), all motifs from motif I to motif II are shown, “row defects” are indicated with yellow dashed lines. For A2O (b), the four best motifs are visualized. For P2O (c), the first 32 motifs lie within 20 meV and can be structured into four different groups. The first occurrence of each group is visualized on the right, while the 40 energetically best motifs of this ranking are visualized in Figure S7.

P2O exhibits multiple different motif candidates within the prediction uncertainty (20 meV). All of them contain the same molecular rows observed in experiment, but our prediction allows for various relative arrangements of these. Figure 5c visualizes the energetic ranking of the 40 best motifs and categorizes them into four different groups. It also visualizes the energetically best motif for each of the four groups, while an exhaustive visualization of all members is given in Figure S7. The first group (colored in blue) has a lower coverage than in the experiment and contains kinks between the molecules. The gaps between rows are a result of the slightly repulsive interactions between the outer molecular rings (compare Figure 3b). The second group (colored in orange) has less space between the molecules, but the coverage is still lower than the experimental one. Group three (colored in green) is the first group with a dense enough packing to be comparable to the experimental results. However, it still contains kinks between subsequent rows. The best motif of this group is energetically 10 meV more favorable than the best densely-packed motif with parallel rows (colored in red), which is well within our prediction uncertainty. This best parallel motif (red motif in Figure 5c) contains a single molecule per unit cell and an area of 125.9 Å^2^/molecule. It is in excellent agreement with the experimentally deduced structure, not only within the STM image (Figure 4d) but also with respect to spot positions and intensities in LEED (Figure 4h). Since the experimental results indicate a point-on-line coincident structure, it is not surprising that the lattice length agrees perfectly for the long axis and deviates only slightly (4%) for the short axis. The enclosed angle agrees within 5° (Table 1). It is presently not clear why, within our calculations, some motifs are energetically more favorable than the best densely-packed parallel motif. The preferability of more loosely packed groups tentatively indicates that our first-principles calculations either slightly overestimate the H–H repulsion or underestimate the intermolecular attraction. Further, it is conceivable that the slight preferability of kinked structures arises from limitations of the exchange-correlation functional, from the dispersion correction method, or from the subtle impact of physical effects that we neglected here (such as finite temperature). At the same time, this energy difference is at the limits of the numerical accuracy (10 meV/molecule for P2O corresponds to approximately 1 μhartree/atom in the calculation), that is, kinked and parallel structures are de facto isoenergetic in our calculations, and this energy difference should not be overinterpreted.

**Table 1:** Comparison of Experimental and Theoretical Unit Cells^a^

	*a*_1_ [Å]	*a*_2_ [Å]	Γ [deg]	θ [deg]	*A* [Å^2^]
B2O,^b^ exp	13.8	11.4	88.5	–14.3	157.4
B2O,^b^ theor	13.0	12.4	94.3	–10.9	160.9
A2O, exp	24.6	24.9	120.0	–36.5	530.0
A2O, theor	24.8	24.8	120.0	–36.6	531.6
P2O, exp	15.0	8.2	95.9	40.8	121.3
P2O, theor	15.0	8.5	100.9	40.9	125.9

a *a*_1_ and *a*_2_, lengths of lattice vectors derived from the epitaxy matrices in combination with the theoretical substrate lattice vectors (primitive lattice constant of Ag(111): 2.842 Å); Γ, angle between lattice vectors; θ, angle between first lattice vector and primitive substrate axis; *A*, unit cell area.

b Data for motif II; for other unit cells, see Table S2.

The experimental preparation of A2O led to a well-ordered, commensurate structure exhibiting a periodic hexagonal pattern. As can be seen in the STM image (Figure 4c), the experimental surface structure is in good agreement with the energetically most favorable theoretical structure containing six molecules per unit cell with an area of 88.6 Å^2^/molecule (see Figure 5b). This cell shows excellent agreement with the predicted structure within our fit uncertainties (Figure 4g). In passing, we note that A2O is the only molecule in our series where we found energetically favorable structures with *C*_3_ symmetry (see details in Supporting Information, section 6).

For B2O, we predict the energetically best motif (labeled motif I) to contain two nonequivalent molecules per primitive unit cell. The molecules in this cell are oriented parallel but located at different adsorption sites, that is, they are (slightly) nonequivalent. The energetically next best structures (2nd to 4th) contain energetically low-lying defects where rows of molecules are rotated by 90°. The structures are visualized in Figure 5a. Within our model, such a defect costs approximately 100 meV per defect to create, while allowing for a denser packing of the B2O molecules. For the sake of discussion, we consider the limiting case where every other row consists of these defects. This structure, which is fourth in our energy ranking and visualized also in Figure 4j, will be called motif II hereafter. It contains four nonequivalent molecules. For comparability, we also use an equivalent cell with four molecules for motif I (Figure 4i). The interpretation of the experimental diffraction pattern (shown in blue in Figure 4f; details are explained in Supporting Information, section 2) indicates that B2O exhibits a line-on-line^52^ registry. Converting that periodicity into real space (Figure 4j) shows that the computed and experimental lattice vectors differ only by −6% and +8%, respectively, while the unit cell areas differ only by 2%. The enclosed angle is reproduced within 6°. We attribute these minor variations to the fact that the experimental structure is line-on-line coincident (compared to A2O, which is commensurate, and P2O, which is point-on-line coincident), while calculations require periodic boundary conditions, which artificially and unavoidably enforce full commensurability between the adsorbate and the substrate.

A summary of the numerical values for the discussed cells is presented in Table 1. More details for all cells shown in Figure 4 are given in Table S2. The agreement between our first-principles structure search and the experimentally found motifs underlines the fidelity of our analysis.

### Influence of Steric Hindrance

**Figure 6:**
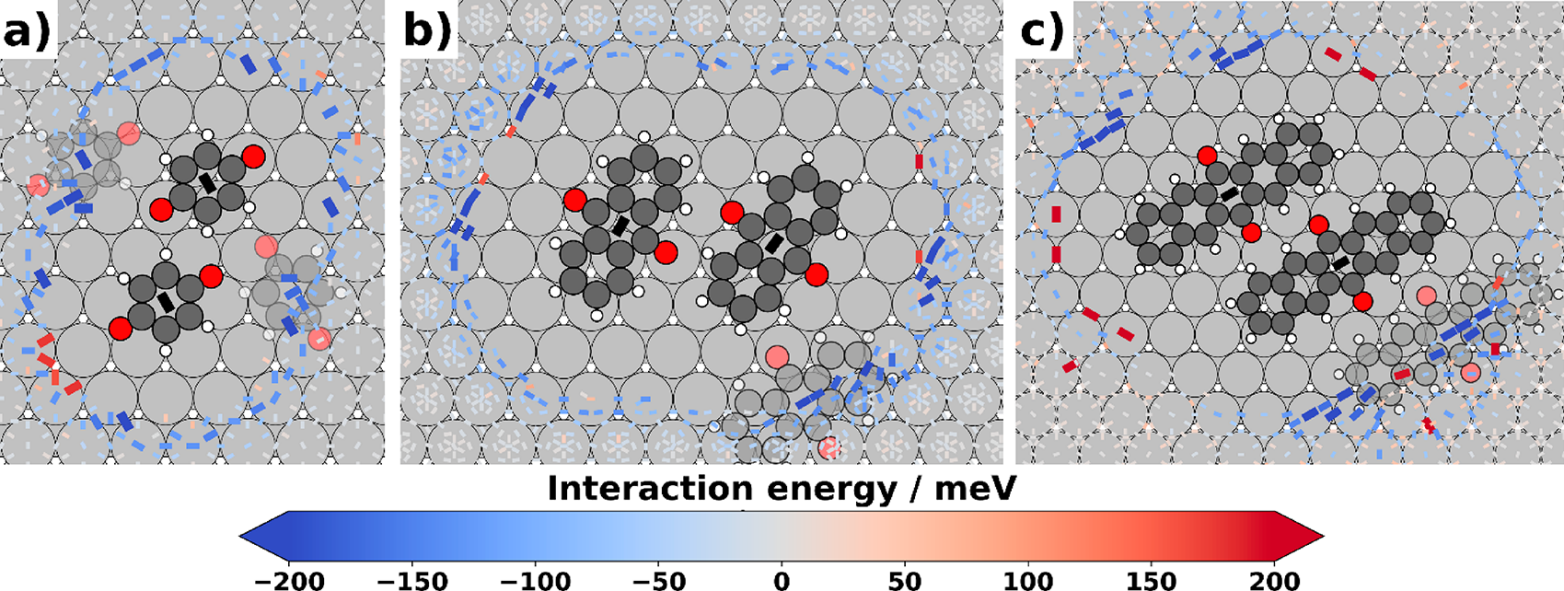
Trimer interactions visualizing all possible interactions of the energetically best pair with an outer molecule for (a) B2O, (b) A2O, and (c) P2O. Each rectangle represents a molecule centered at the rectangle’s midpoint, with its backbone orientation indicated by the long axis of said rectangle. The respective rotation indicates the energetically most favorable third molecule at this specific point, all other rotations at each site were discarded for clarity. The rectangles are scaled according to their absolute energy value to highlight strong interactions. The semitransparent molecules illustrate energetically favorable positions for the third molecule.

We have shown earlier in this work that the pairwise interactions as well as the molecule–surface interactions are similar for all three different systems. Nevertheless, the motifs observed experimentally and predicted theoretically exhibit substantially different features. This shows that there must be a crucial, hitherto missing, factor influencing the packing motifs formed. This factor is the steric hindrance between molecules. To illustrate and quantify this effect, we took the energetically most favorable pair for each system and evaluated the interactions to a third molecule at different orientations and positions. The resulting visualization (Figure 6) is similar to that in Figure 3b, but now the orientation of the outer molecule is visualized *via* the orientation of a rectangle. All rectangles were colored according to their energy. Furthermore, the size was scaled according to their absolute energy to focus on stronger interactions. Note that here the energy range is ±200 meV to focus on the strongest trimer interactions. The visualization reveals that B2O can arrange such that the energetically most favorable interactions in all directions can be exploited for monolayer formation, leading to highly attractive interactions in four directions for each molecule. A2O can build structures with molecular triangles, which also allow highly attractive interactions per molecule, as can be seen on the lower right corner of Figure 6b. For P2O, the conjugated backbone is so long that the attractive hydrogen–oxygen interactions can only be formed with two neighbors. This makes interactions involving molecules rotated relative to each other energetically much less favorable, leading instead to long rows of molecules aligned parallel to each other.

### Evaluating the Driving Forces

**Figure 7:**
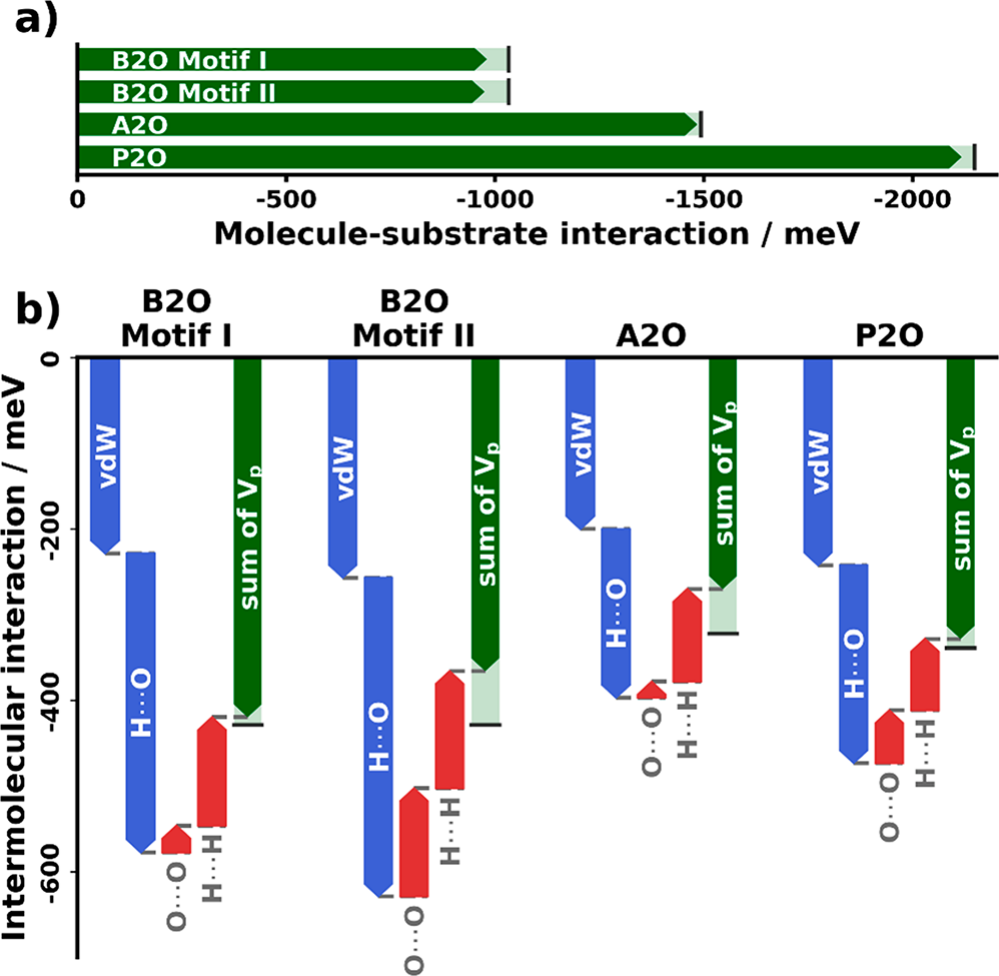
Detailed breakdown of interaction energies for the motifs formed by the three molecules (Figure 4i–l) into van der Waals (vdW) and electronic interactions between molecular fragments. (a) Molecular adsorption energy. (b) Total intermolecular interaction energies per molecule separated into contributions of vdW and molecular fragments. The green arrows indicate the resulting interaction energy (which is equivalent to the sum of all *V_p_* (see eq 1) for a motif candidate). The green shaded areas indicate the hypothetically best possible values for the sum of contributions if they could be realized separately, which is not possible as real motifs are always a trade-off between molecule–substrate and intermolecular interactions.

With all driving forces at hand, it is possible to quantitatively discuss the energetic contributions for the motifs formed by each molecule (Figure 4i–l). Figure 7a shows the mean molecule–substrate interactions of the observed motifs compared to the corresponding best possible molecule–substrate interaction for a single molecule on the surface. In Figure 7b, we visualize the total intermolecular interaction energies (*i.e*., the sum of all pairwise interaction energies present in the motif). There, we compare the intermolecular interactions for the observed motifs (green arrows) to the hypothetically best possible intermolecular interactions obtainable when fully neglecting molecule–surface interactions (shaded areas).

As a first step, the energy decomposition now allows us to briefly reconsider the energetic differences between the two B2O motifs we compared to experiment. For both motifs, the molecule–substrate interactions are roughly the same when averaged over all comprising adsorption geometries. The energetic difference is the result of intermolecular interactions. Thus, within the commensurate model, motif I is the favored structure in terms of *effective* energies (green arrows in Figure 7b). The decomposition, however, shows that van der Waals and oxygen–hydrogen interactions (*cf.* blue arrows) are slightly more favorable for motif II. Yet, its effective intermolecular energy is less favorable than that of motif I due to the stronger oxygen–oxygen repulsion, marked by red arrows. The energetic difference between motifs I and II might decrease or even vanish if motif II could be calculated as a truly line-on-line coincident structure, which would allow it to decrease O–O repulsion by slightly increasing the distance between rows with different molecule rotation. While *ab initio* methods are currently not able to predict fully incommensurate structures (or those for which the commensurate supercell would be intractably large), our energy decomposition here allows us to anticipate the implications of slight incommensurability.

We can now continue with a comparison of the driving forces for the three different molecules. For A2O, the observed motif (Figure 4) includes only the energetically best adsorption geometries, while the corresponding motifs for B2O and P2O also contain worse-ranked local geometries, resulting in a small adsorption energy penalty. This penalty is compensated by the larger intermolecular interaction energies those two systems can realize compared to A2O. While for all four motifs the role of van der Waals interactions is similar, the total electronic contributions of the fragments (*i.e.*, interactions with all neighboring molecules) are smaller for the larger molecules. This is caused by a reduced availability of highly attractive intermolecular interactions due to steric hindrance. The interaction energy for the best motif of A2O is lower than that for P2O due to the trade-off between molecule–substrate and intermolecular energies it needs to take. This could be reformulated into a design principle: *For molecules to form structures dominated by intermolecular interactions, the energy to be gained by intermolecular interactions must be larger than the energy penalty when placing a molecule in an adsorption geometry that is not the global minimum.*

This condition is fulfilled for the largest molecule in the series, P2O, due to the high number of energetically favorable adsorption geometries and also for the smallest molecule, B2O, due to the highly favorable intermolecular interactions. However, it is not fulfilled for the intermediate-sized A2O, where the molecule–surface interaction is more prominent than the intermolecular driving forces. One could *a priori* assume that, if such a condition is fulfilled for some molecules with a given functionalization, it would also be fulfilled for others with the same functionalization. Conversely, here we see that this is not even the case for a homologous series, where investigating the adsorption behavior of the smallest (B2O) and largest (P2O) molecule would not allow us to deduce the adsorption behavior for the intermediate-sized molecule (A2O).

## Conclusion

In this work, we addressed the question of why molecules with identical functionalization form completely different structural motifs on a metal surface and whether insights and general trends for the on-surface self-assembly mechanisms can be retrieved from first-principles without prior experimental input. To understand this behavior and reveal general trends, we investigated all the interaction mechanisms involved.

We find that the molecular functionalization is responsible for the dominant intermolecular interactions and their magnitude, as well as the adsorption sites that are generally accessible on the substrate. The size and shape of the backbone, on the other hand, determines the number of these intermolecular interactions each molecule can realize, together with how easily (in terms of energy) different adsorption sites on the substrate are accessible.

For all molecules, the motif that forms in experiment is then a trade-off between these antagonizing effects. The intermolecular interaction tends to be the dominant factor when it is strong *and* readily accessible, either because the backbone is small (allowing many attractive intermolecular interactions) or when the energetic difference between adsorption positions is small, allowing molecules to trade the best adsorption site for an energetically more beneficial intermolecular interaction. For “intermediate” sized backbones, the impact of the substrate can overpower those intermolecular interactions, leading to a motif dominated by the best adsorption positions. This generalized formulation is consistent with our findings for acenequinones on Ag(111):

The compact structure of B2O allows four oxygen–hydrogen interactions per molecule. These interactions lead to a brick-wall structure. For A2O, the larger backbone favors the formation of triangular building blocks, which, in combination with highly beneficial adsorption geometries, lead to the observed hexagonal ring-like motif. The even larger P2O molecules can only interact favorably with two neighboring molecules. The anisotropy of these interactions leads consequentially to the parallel alignment of P2O, thereby forming molecular rows. Our findings are backed by excellent agreement between theoretically predicted structures and experimentally resolved packing motifs.

These insights showcase that the vastly different surface patterns are driven by a changing balance of surface–molecule and intermolecular interactions in combination with steric hindrance upon increasing the molecule size. Even for relatively simple systems like a homologous series of quinones, it is therefore challenging, if not outright impossible, to predict or engineer the monolayer structures based on simple design principles alone. On a more positive note, advanced computational tools based on machine-learning, such as our SAMPLE^45^ approach, allow one to retrieve quantitative interaction energies and extract general trends for the interaction mechanisms. With enough structures investigated, we expect this to be a helpful step toward a more holistic design of surface structures.

## Methods

All calculations were performed with the FHI-aims package.^53^ We combined the exchange-correlation functional PBE^49^ with the TS^surf^ correction^47,48^ to account for long-range dispersion interactions. The PBE+TS^surf^ combination is very robust, widely used, and known to give very accurate results, both for the electronic structure^54^ and for adsorption energies.^55−58^ The integration in k-space was performed with a Γ-centered grid with a well converged density of 36 points per primitive lattice direction and one k-point in *z* direction. As our calculations involved unit cells with different shapes, the k-points were scaled according to the length of the unit cell vectors. The periodic nature of our systems allowed us to use the repeated slab approach with a unit cell height of 80 Å (including >50 Å of vacuum), a dipole correction,^59^ and eight layers of Ag with a mixed-quality numerical basis set (details in Supporting Information, section 1.1). With this approach, all adsorption energies were converged to a methodological uncertainty below 20 meV per adsorbate molecule.

Finding all local minima for the molecules on the surface would, in principle, require an exhaustive global structure search. This is infeasible even for the most advanced algorithms^60,61^ due to the high configurational complexity.^42^ Thus, we performed a two-step procedure that starts by first optimizing a single molecule on the surface and consecutively utilizing the BOSS approach^61^ to find all local extrema in the three-dimensional (*x*, *y*, and rotation around molecular axis) PES. As a second step, geometry optimizations were performed from all extrema in the aforementioned PES where the whole molecule and the two topmost metal layers were allowed to relax. All final adsorption geometries for which at least one atom position of the molecule differed by more than 0.1 Å (with symmetries taken into account) were considered as separate adsorption geometries (see also Supporting Information, section 1.2).

We define the adsorption energy as *E*_ads_ = *E*_sys_ – *E*_sub_ – *E*_mol_, where *E*_sys_ is the energy of the combined system, *E*_mol_ is the energy of a molecule in the gas phase, and *E*_sub_ is the energy of the pristine Ag slab with the two upper layers prerelaxed. Negative values of *E*_ads_ denote energy gain upon adsorption.

The SAMPLE^45^ approach takes the surface atom positions in a given unit cell as a discrete grid and generates all combinations of building blocks at all possible positions within the unit cell and then removes colliding structures. As building blocks, it uses all adsorption geometries with all their symmetry equivalents on the respective metal surface. To not only be limited to a single unit cell, with SAMPLE we also generate an exhaustive set of unit cells for a given unit cell size (number of substrate atoms). For all three molecules in this study, we varied the unit cell sizes and number of molecules per cell to ensure that experimentally feasible motifs are part of the prediction set (details in Supporting Information, section 1.3). The training set for the SAMPLE approach was chosen with experimental design employing the D-optimality criterion^46^ on interactions in the motifs. For the description of the different configurations within SAMPLE, the species dependent feature vector, considering distances between hydrogens and oxygens in all combinations, was used. All hyperparameters were thoroughly converged to robust values (Supporting Information, section 1.4). To further reduce the computational costs, we first calculated the training points as free-standing monolayers (i.e., removed the metal substrate) and used the resulting fit parameters for the pairwise interaction energies as priors for the on-surface systems. With this methodology, we could reduce the number of needed training calculations to 249 for B2O, 245 for A2O, and 82 for P2O.

When estimating the uncertainty of a trained machine learning model, a standard approach is to use a separate validation set (holdout). This approach works well when data generation is cheap (big data) and large amounts of training data are needed. In our case, each data point for the machine learning model is a noticeable investment, and therefore, all the data should be exploited as far as possible. Thus, we instead use leave-one-out cross validation^62^ to estimate the uncertainties of the model. For this, we train the model on all data points but one and calculate the error for this point (difference between prediction and DFT energy). We repeat this process for all points in the training set and then calculate the root-mean-square error of all those energy deviations. Our training points are chosen D-optimally, meaning that the most important points should be calculated. Therefore, in our case this approach, to some extent, is a worst-case measure of the uncertainty, as, if there are important data points in the set, the uncertainty for those will be very large, increasing the overall uncertainty. For testing the accuracy of SAMPLE predictions, we consider the LOOCV-RMSE uncertainty superior to simple RMSE evaluation on a separate test set, additionally saving the costs of calculating an expensive test set. The LOOCV-RMSE uncertainties obtained for our three model systems are 11 meV for B2O, 9 meV for A2O, and 20 meV for P2O.

The fully trained energy models were then used to predict the energies and rank the full set of possible motifs. Details of this prediction process can be found in Supporting Information, section 3.

The interaction energies of adsorption-induced dipoles with their periodic replicas were estimated by summing over all dipole interaction energies via . Here, *r*_*i*_ is the distance between the central unit cell and each of its neighbors, and μ is the effective point dipole of the unit cell. The sum over unit cells is performed until the energy is converged to below 10^–4^ eV.

To compare the theoretical structure to LEED results, simulations based on kinematic diffraction theory were performed. Therefore, the location and intensity of the peaks were calculated as the square of the structure factors . Here  are the reciprocal lattice vectors of the crystal and  are the positions of atoms in the unit cell. The atomic form factors were each approximated with  where *a*_*i*_ and *b*_*i*_ were taken from the International Tables for Crystallography (2006).^63^

The organic molecules B2O (CAS 106-51-4, nominal purity 99.5%), A2O (CAS 84-65-1, nominal purity 97%), and P2O (CAS 3029-32-1, nominal purity 99%) were obtained as powders from Sigma-Aldrich. A2O and P2O could be further purified by temperature gradient vacuum sublimation using a CreaPhys DSU-05 apparatus. B2O was purified in a home-built sublimation device consisting of two separate glass tubes, dubbed reservoir and sublimation tube, which are connected to each other by an angle valve, with the sublimation tube additionally attached to the deposition chamber by a dosing valve. B2O was initially filled into the reservoir tube and evacuated with both valves open. The reservoir tube was subsequently heated by a stream of hot air until a sufficient amount of B2O deposited on the walls of the sublimation tube, after which both valves were closed.

The Ag(111) single crystals were obtained from MaTeck GmbH and cleaned by Ar^+^ sputtering at 700 eV and incident angles of ±45° to the surface normal, followed by annealing at 800 K. Sputtering and annealing were cyclically repeated until the surface quality was satisfactory, as confirmed by low energy electron diffraction (LEED).

The monolayers were deposited at room temperature (296 K) in an ultrahigh vacuum chamber with a base pressure better than 5 × 10^–10^ mbar via physical vapor deposition. B2O and A2O were deposited directly from the gas phase due to their high vapor pressure at room temperature.^64,65^ The deposition of B2O was carried out by positioning the sample at approximately 30 cm distance and in direct line of sight to the dosing valve and opening it until the chamber pressure reached 1 × 10^–6^ mbar, after which the valve was kept open for 10 minutes. For the deposition of A2O, the purified powder was filled into another glass tube and connected to the deposition chamber *via* a dosing valve. During layer deposition, the sample was placed at approximately 30 cm distance and in direct line of sight to the dosing valve, and the dosing valve was opened for 15 min. P2O was deposited by thermal evaporation from a shutter-controlled effusion cell held at 450 K with a deposition time of 10 min. When we increased the deposition time for P2O, we were able to create films thicker than one monolayer. For B2O and A2O, only the first and typically most strongly bound molecular layer forms well-ordered structures, while higher layers desorb from the surface due to the thermal energy at room temperature. Therefore, no further annealing was attempted for those samples. In the case of P2O, after deposition the samples were gently heated while being monitored by LEED until a well-ordered structure became visible.

For the quantitative structural analysis, the samples were first characterized by distortion-corrected LEED^66^ at room temperature including a numerical fitting of the assumed surface unit cell in reciprocal space to the measured LEED pattern (LEEDLab 2018, version 1.4). After LEED examinations, the samples were transferred into a low-temperature scanning tunnelling microscope (SPECS JT-LT-STM/AFM with Kolibri Sensors) and measured in constant-current mode at 4.5 K for A2O and 1.2 K for B2O and P2O. Afterward, the obtained STM images were subjected to a two-dimensional Fourier transform, and the epitaxy matrices were determined from those results (details in Supporting Information, section 2), utilizing the same software tools that were used for the LEED measurements, as already described elsewhere.^67−69^

In the P2O case, one structure emerged during annealing. Afterward no changes to the structure occurred during measurement at room temperature or low temperature over a prolonged period of time. A2O showed a LEED pattern with a single structure at room temperature after deposition. During the cooling process to 4.5 K, a second structure with hexagonal symmetry and lower coverage (which is also discussed in the main text) emerged, sometimes accompanied by various other structures. From the emerging structures, said hexagonal structure was among the most abundant ones. This implies, tentatively, that the other emerging structures are kinetically trapped. Similarly, we also interpret the still-present high-coverage structure as kinetically trapped. Thermodynamically, shifting to lower temperatures would systematically favor higher-coverage structures.^70^ The fact that the lower-coverage structure only emerges during cooling, therefore, tentatively implies that the high-coverage phase observed after deposition is not stable at measurement conditions but has not had enough time to fully convert yet. We therefore used the hexagonal structure for further comparison in this manuscript. For B2O also only a single structure was visible in LEED that did not change upon cooling down to 4.5 K. All structures were stable during STM measurements.

## Supporting Information Available

Theoretical methods and the prediction process, an extensive comparison of the unit cells mentioned in the manuscript, the fit procedure for the A2O unit cell, supplementary analyses of the intermolecular interactions at different molecular rotations and the influence of symmetry (PDF)

## Notes

The calculations used in this manuscript are available *via* the NOMAD database (www.nomad-repository.eu) as separate data sets for B2O (https://dx.doi.org/10.17172/NOMAD/2021.03.09-3), A2O (https://dx.doi.org/10.17172/NOMAD/2021.03.09-2) and P2O (https://dx.doi.org/10.17172/NOMAD/2021.03.09-1). Experimental details are provided in the Supporting Information; further experimental data is available upon request.
