# Supplementary material for: Nonintuitive Surface Self-Assembly of Functionalized Molecules on Ag(111)
Source: ACS Nano. 2021 Mar 17;15(4):6723–34. doi: 10.1021/acsnano.0c10065 (PMC8155339; doi:10.1021/acsnano.0c10065)
Supplement: Supplementary file 1 — nn0c10065_si_001.pdf [file nn0c10065_si_001.pdf]

Supporting Information to “Nonintuitive Surface Self-Assembly of Functionalized Molecules on Ag(111)”

A. Jeindl[1], J. Domke[2], L. Hörmann[1], F. Sojka[2], R. Forker[2], T. Fritz[2], and O.T. Hofmann[1*]

[1] Institute of Solid State Physics, NAWI Graz, Graz University of Technology, Petersgasse 16, 8010 Graz, Austria

[2] Institute of Solid State Physics, Friedrich Schiller University Jena, Helmholtzweg 5, 07743 Jena, Germany

*Corresponding author: o.hofmann@tugraz.at

Content

1 Supporting Methods for Theory 2

1.1 Mixed Basis Set Approach 2

1.2 Finding Adsorption Geometries 3

1.3 Generating Motif Candidates 3

1.4 Hyperparameters Used for SAMPLE 3

1.5 Checking the Interaction Energies 4

2 Details of the FFT Fit Procedure 5

3 Predictions of All Possible Motifs 8

4 Unit Cell Comparison 10

5 Additional Pair Interaction Information 12

6 Symmetry Considerations of the Predicted Structures 13

1 Supporting Methods for Theory

1.1 Mixed Basis Set Approach

A crucial part in surface slab calculations is the convergence of the number of slab layers. There need to be enough layers below the surface to resemble bulk-like behavior, but as few as possible to reduce computational cost. In our case, more than 6 layers of silver are necessary for a sufficiently converged adsorption energy, which increased the computational cost to an intractable level. Fortunately, a fully accurate quantum mechanical description is only necessary for the interaction area of a molecule and the substrate. Thus, the fact that FHI-aims[1–5] uses atom-centered basis sets was utilized in the following way: The uppermost layers of the surface slab were calculated with a tight and thoroughly converged basis, while the lower layers (which are further away from the molecule) were only represented *via* a very light basis (see Figure S1c).

The numerical influence of exchanging metal layers with “cheap” atoms is visualized in Figure S1 for the adsorption energy of the best adsorption positions of B2O. The relevant property determining the uncertainty in relative stability of our motifs is the energetic difference between adsorption geometries. The convergence of this property depending on the number of layers for a fully tight and a mixed basis set is visualized in Figure S1a. To visualize the influence of exchanging lower lying layers with “cheap” atoms, we started with a full slab and gradually exchanged layers by “cheap” atoms. This is shown in Figure S1b. Based on the convergence tests we used 8 layers of silver with three tight layers and 5 “cheap” layers underneath. A visualization of this with a molecule on top is shown in Figure S1c.

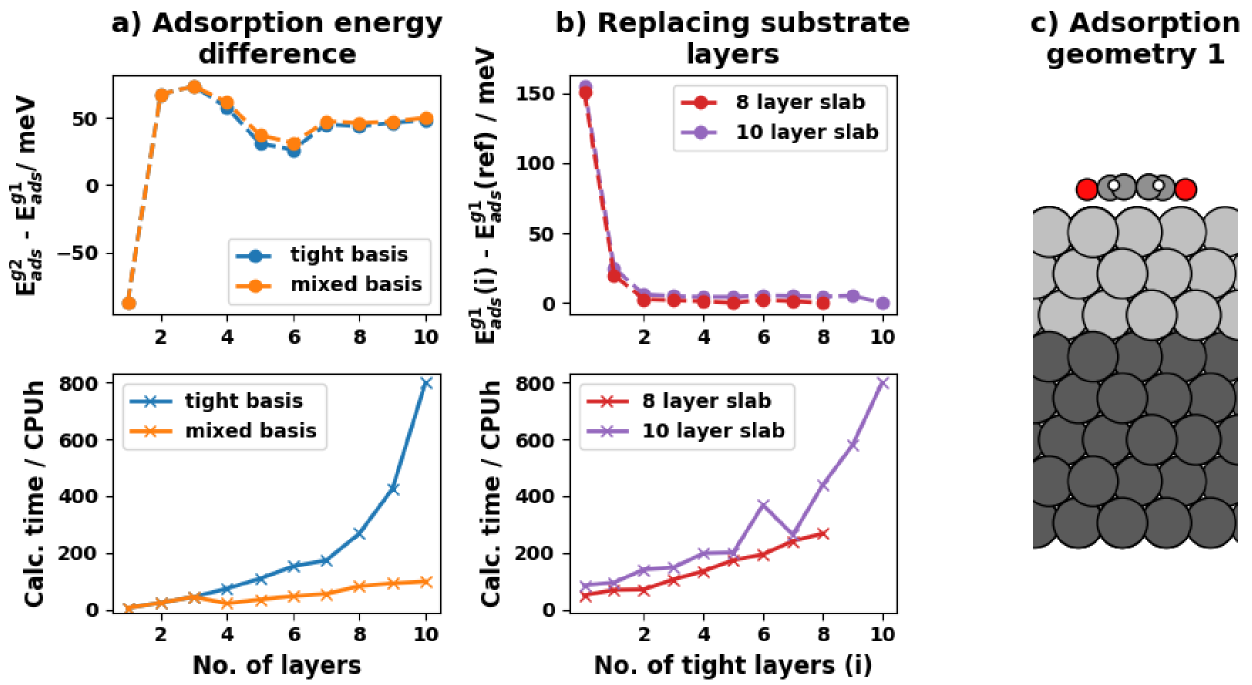

Figure S1: a) Energy difference between adsorption geometry 1 and 2 of B2O in dependence of the number of substrate layers. For the mixed basis set, the first three layers were computed with “tight” settings and the subsequent layers were described using “cheap” Ag settings; b) Adsorption energy of adsorption geometry 1 when subsequently replacing “tight“ Ag layers with “cheap” ones; c) Visualization of the best local adsorption geometry of B2O adsorbed on the mixed basis used in this manuscript. The light grey layers consist of Ag atoms with tight basis while the dark grey atoms were calculated with a much looser basis.

1.2 Finding Adsorption Geometries

Gaussian process regression[6] was used to interpolate the adsorption geometries of single molecules on a multidimensional potential energy surface, in the following way: First, an individual molecule was optimized lying flat on the surface (as was also found experimentally for P2O)[7] at an unspecific in-plane position to estimate the general distortions of the molecule upon adsorption on a surface. This optimized molecule was symmetrized and lifted from the surface by 0.1 Å to reduce the influence of the Pauli repulsion, *i.e.*, to avoid "ramming" the molecule into the substrate. The lateral position of minima is not affected by the slightly larger vertical distance. This approach resulted in a mean distance between the center of the uppermost Ag layer and the molecular backbone of 2.4 Å for B2O, 2.9 Å for A2O and 3.0 Å for P2O. Then, a full potential energy surface (PES) for movement of this molecule along the x-y plane and rotation around the z-axis was mapped by performing approx. 50 DFT calculations. Subsequently, a geometry optimization for the full molecule and substrate was performed from all extrema in the aforementioned PES, allowing the whole molecule to relax until the remaining forces were below a suitable threshold for all atoms (0.02 eV/Å for A2O and P2O, 0.05 eV/Å for B2O). Those adsorption geometries were then symmetrized according to the applicable substrate symmetries to remove geometry-optimization artefacts. All final adsorption geometries, where at least one atom position differed by more than 0.1 Å (with symmetries taken into account), were considered as separate adsorption geometries.

1.3 Generating Motif Candidates

For all three molecules in this study we varied the number of molecules per cell N_A from 1 to 4 and varied the unit cell size from $N_A * A_{\mathrm{min}}$ to $N_A * (A_{min} + 5)$ to ensure that experimentally feasible configurations are part of the prediction set. Here A_{min} is the minimal cell size where configurations could be built without interfering with minimal distance thresholds set in SAMPLE.
For B2O and A2O we additionally constructed tightly packed polymorphs with up to 6 molecules per unit cell. The hexagonal cell for A2O was found by constructing all configurations for hexagonal unit cells with six molecules per unit cell up to 90 surface atoms per cell.

1.4 Hyperparameters Used for SAMPLE

For the SAMPLE approach[8] several hyperparameters are necessary. All of those hyperparameters were varied systematically to maximize the log-likelihood in the Bayesian linear regression formalism. Supplementary Table 1 contains all optimized hyperparameters used for the prediction of the three systems. Decay length differences arise from the different molecule sizes. To avoid the need to fit the highly repulsive Pauli repulsion region with Bayesian linear regression, SAMPLE uses a minimal distance threshold for all atom-species combinations. For this work the following thresholds were used: O↔H: 1.6 Å; O↔O: 2.4 Å, H↔H: 1.6 Å; C↔H: 2.3 Å; C↔O: 2.5 Å.

Table S1: Hyperparameters of the SAMPLE approach used for the structure prediction of benzo-, anthra- and pentacenequinone.

Hyperparameter	B2O	A2O	P2O
Adsorption energy uncertainty	100 meV	100 meV	100 meV
Interaction energy uncertainty	300 meV	300 meV	300 meV
DFT data uncertainty	10 meV	5 meV	5 meV
Decay length	5 Å	5 Å	10 Å
Decay power	3	3	3
Decay length feature space	12	9	5
Feature threshold	0.0075	0.0075	0.01

1.5 Checking the Interaction Energies

It is unfortunately not possible to directly check the mapped fragment energies against *ab initio* energies. To get an idea of the accuracy of the mapping we performed the following test for our mappings: First we check the pairwise interaction energies of the fully trained system with the separately trained pair energies for the van der Waals and electronic contributions (Figure S2a). Here the maximum error between the full system and the sum of contributions is below 1 meV. As a second step we compare the electronic energy with the pairwise interaction energies that were mapped onto separate fragments (Figure S2b). Here the accuracy is slightly worse, but the largest difference between interaction energies is still only 10 meV.

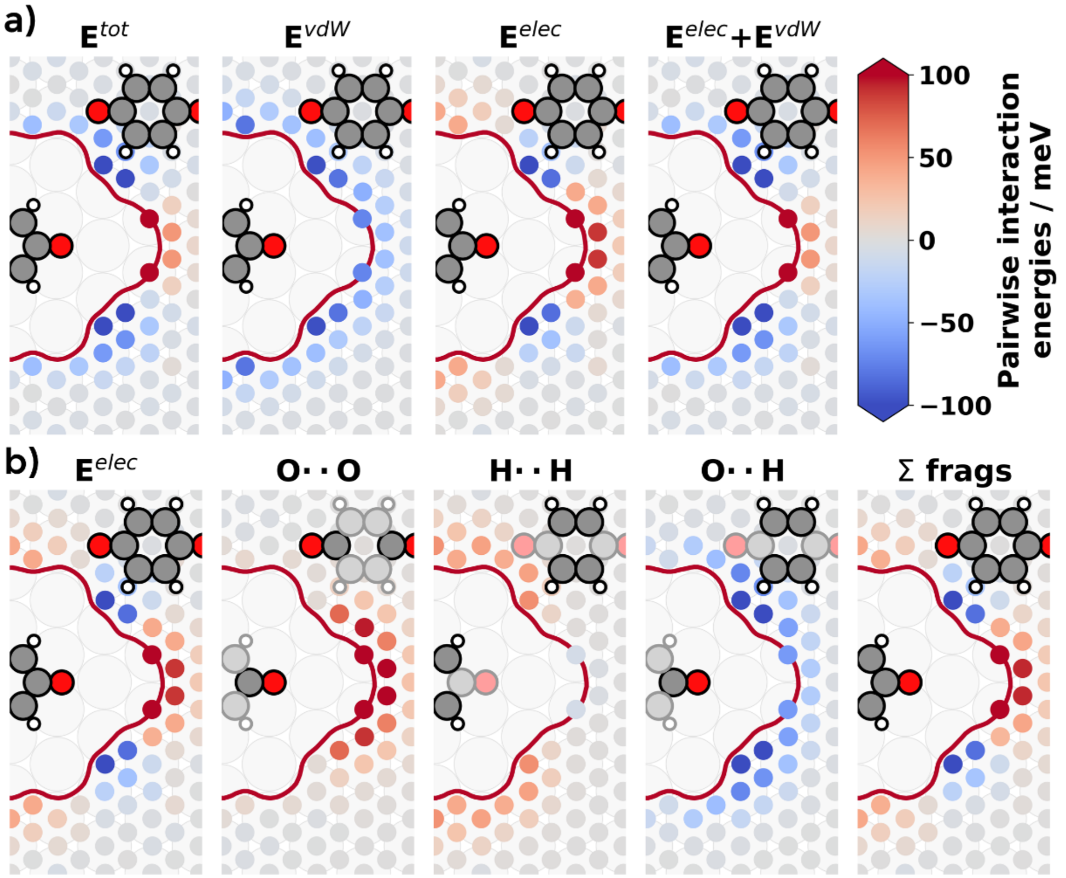

Figure S2: a) Pairwise energies when training the total energy (left) compared to the sum of electronic and van der Waals energies (right); b) Pairwise energies when training with the electronic energy only (left) compared to the sum of the trained fragments (right)

2 Details of the FFT Fit Procedure

Since the LEED device used by us typically probes a surface area in the order of 1 mm^2, the resulting images usually show a superposition of all motifs present in that area, *e.g.*, symmetrically equivalent domains and other polymorphs, if present. For this reason, we additionally performed a detailed analysis of the STM images to ensure the assignment of the epitaxial relations determined by LEED to the motifs presented in STM as well as to increase the accuracy. We found motifs exhibiting Moiré patterns in their respective STM images on all samples presented in this study. These patterns show up as discrete spots in the two-dimensional Fourier transforms (FFT) as well, and their spot positions can be described in the same way as multiple scattering in geometric scattering theory, thus enabling an analysis[9] with LEEDLab[10]. For that purpose, we subjected the STM images showing a single domain of the motif under investigation, featuring molecular resolution as well as a Moiré pattern, to an FFT. We then determined the epitaxial relation by fitting the respective reciprocal lattice including the Moiré spots to the FFT, optimizing the adsorbate lattice and substrate lattice simultaneously, thus circumventing the distortions typically present in STM. This procedure yielded the epitaxy matrices for B2O and P2O directly (see Figure S3). However, it is not applicable to the hexagonal A2O structure, where we observed no Moiré patterns (likely due to its commensurate registry). Instead, we analyzed an STM image containing the motif presented in the main text (Motif A) and an additional, non-commensurate motif (Motif B), which shows a Moiré contrast, as can be seen in Figure S4. By taking a detail of the STM image containing only Motif B, we determined its lattice vectors relative to the substrate vectors, which then contain the local distortion, using the procedure described earlier. We presume the distortions to be uniform for the image as a whole and therefore particularly for a detail containing only Motif A and use this detail to determine the lattice vectors of Motif A relative to Motif B, this time solely optimizing the adsorbate lattice. We then calculated the epitaxial relation of Motif A relative to the substrate by combining the two derived epitaxy matrices *via* matrix multiplication. By comparing both obtained motifs in reciprocal space to the LEED image of the A2O sample (Figure S5), it is evident that a structure determination based on LEED alone would probably remain ambiguous due to the similarity in many spot positions. Motif B is likely a kinetically trapped structure and could thus, and due to its non-commensurate nature, not be found within the theoretical framework (For details see Supporting Information, section 3).

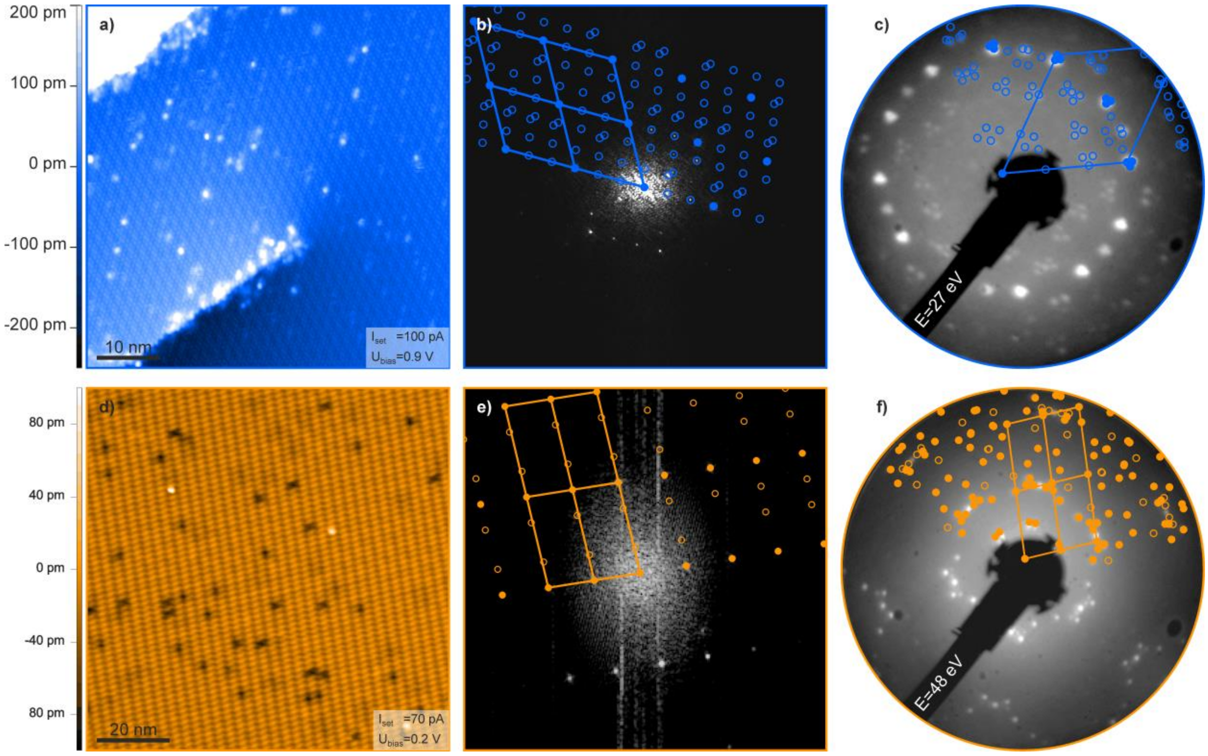

Figure S3: STM images of a) B2O (blue) and d) P2O (orange) (same sample as the images in Figure 4) b,e) FFTs of a) and d), respectively, superimposed with the fitted reciprocal lattice. Several lattice points (dots) as well as Moiré frequencies (circles) are highlighted. c,f) LEED images (same measurement as Figure 4) at a given primary electron energy E superimposed with a simulation of the reciprocal lattice (dots) including multiple scattering (circles) and symmetrically equivalent domains as fitted to (b) and (e), respectively.

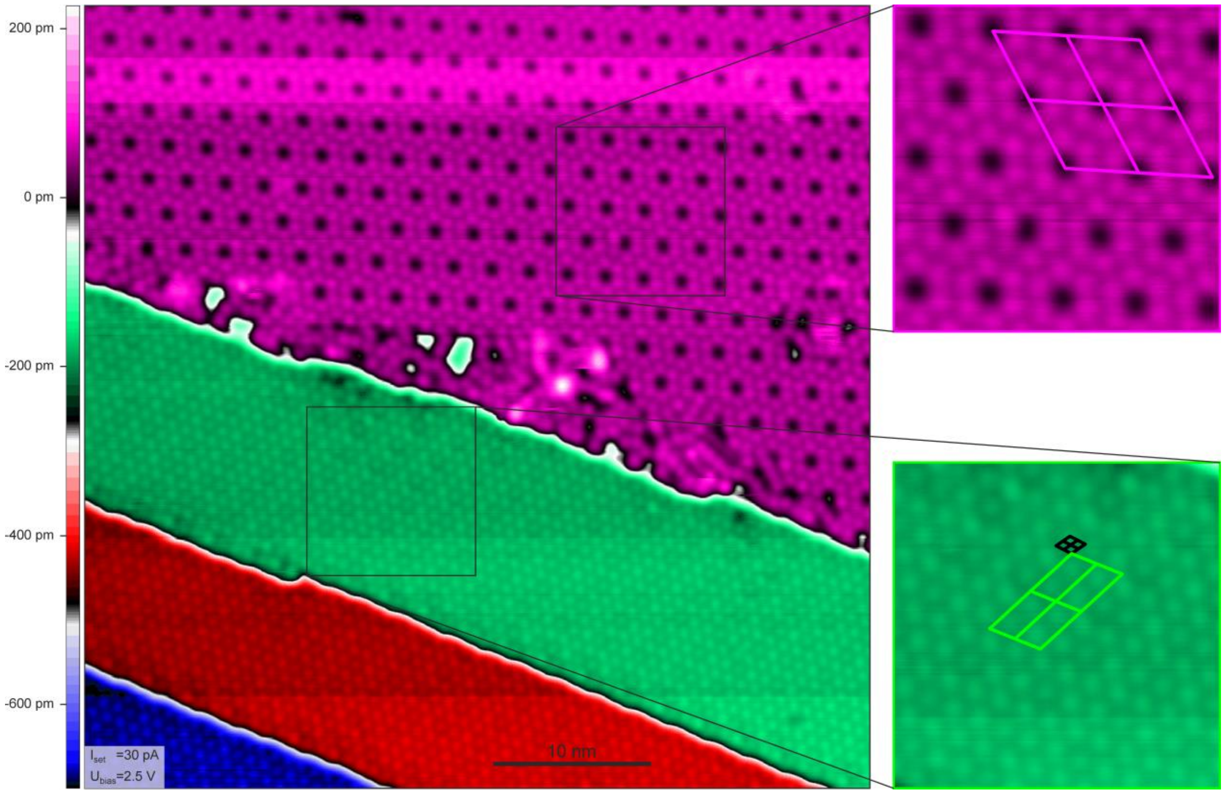

Figure S4: STM image used for the structure analysis of A2O. Details of Motif A (top, magenta color code) and Motif B (bottom, green color code) feature representations of the unit cells of the motifs fitted to an FFT of the respective detail. The substrate lattice characterizing the distortions present in the image as determined by the fit is shown in comparison to Motif B (black lattice cells).

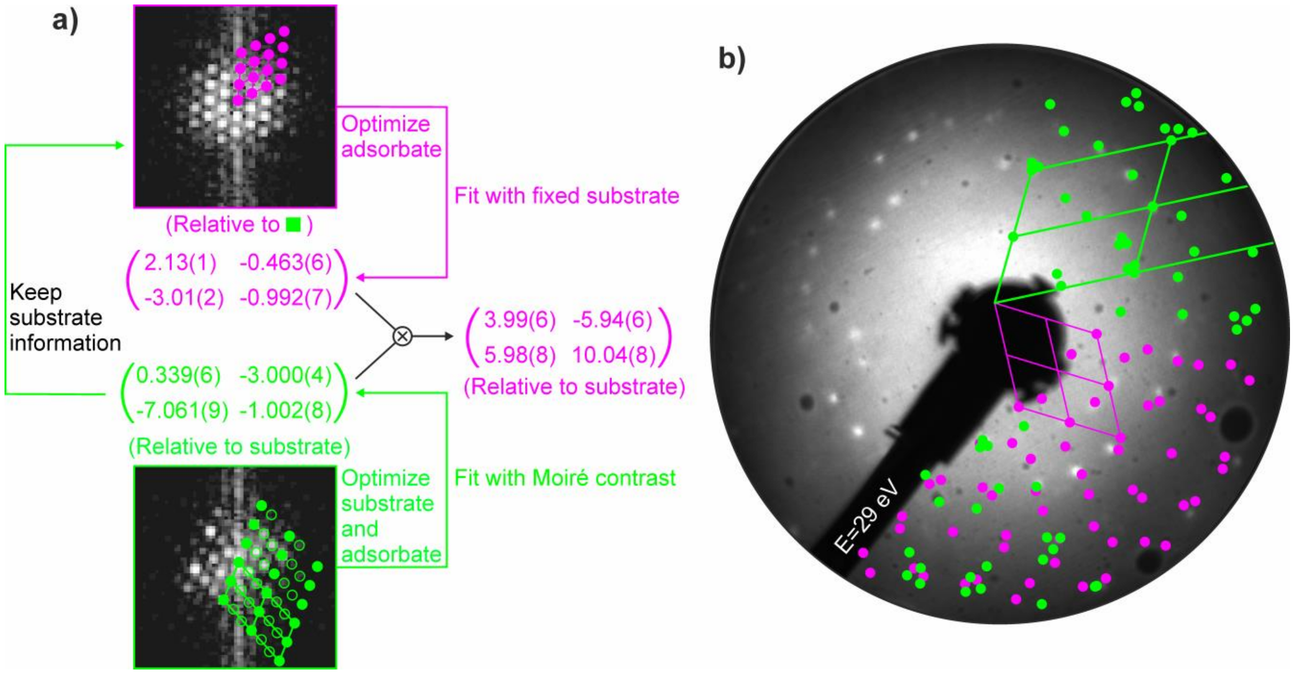

Figure S5: Reciprocal space representation of the lattices of Motif A (magenta) and Motif B (green). a) FFTs of the details displayed in Figure S4 superimposed with the reciprocal unit cell as fitted to the FFT including the respective epitaxy matrix. Several reciprocal lattice points (filled circles) as well as Moiré spots (open circles) for Motifs A and B are displayed as guide to the eye. b) LEED of the sample measured at 296 K, superimposed with a simulation of the reciprocal lattice points including symmetrically equivalent lattice representations.

3 Predictions of All Possible Motifs

To obtain the motifs discussed in the main paper, first all possible motifs within the model discretization were created (see Supporting Information, section 1.4) to then predict all their energies. The energy models used for prediction were trained on a representative number of D-optimally chosen calculations (249 for B2O, 245 for A2O and 84 for P2O). To speed up training, intermolecular interaction data obtained from free-standing monolayers (*i.e.*, removed substrate) was used as prior for the on-surface models. The prediction results for the best motifs are already shown in Figure 4. Figure S6 visualizes the full ranking of all motifs generated for each molecule according to energy per molecule.

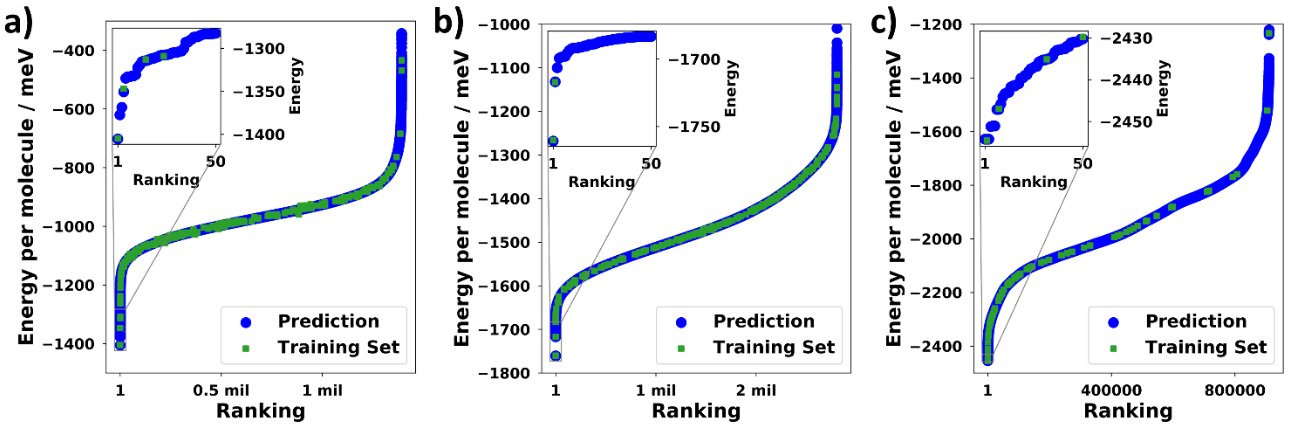

Figure S6: Ranking of all predicted motifs according to formation energy per molecule (from most favorable to least favorable) for a) B2O, b) A2O, and c) P2O. Points used for the training of the respective models are visualized with green rectangles.

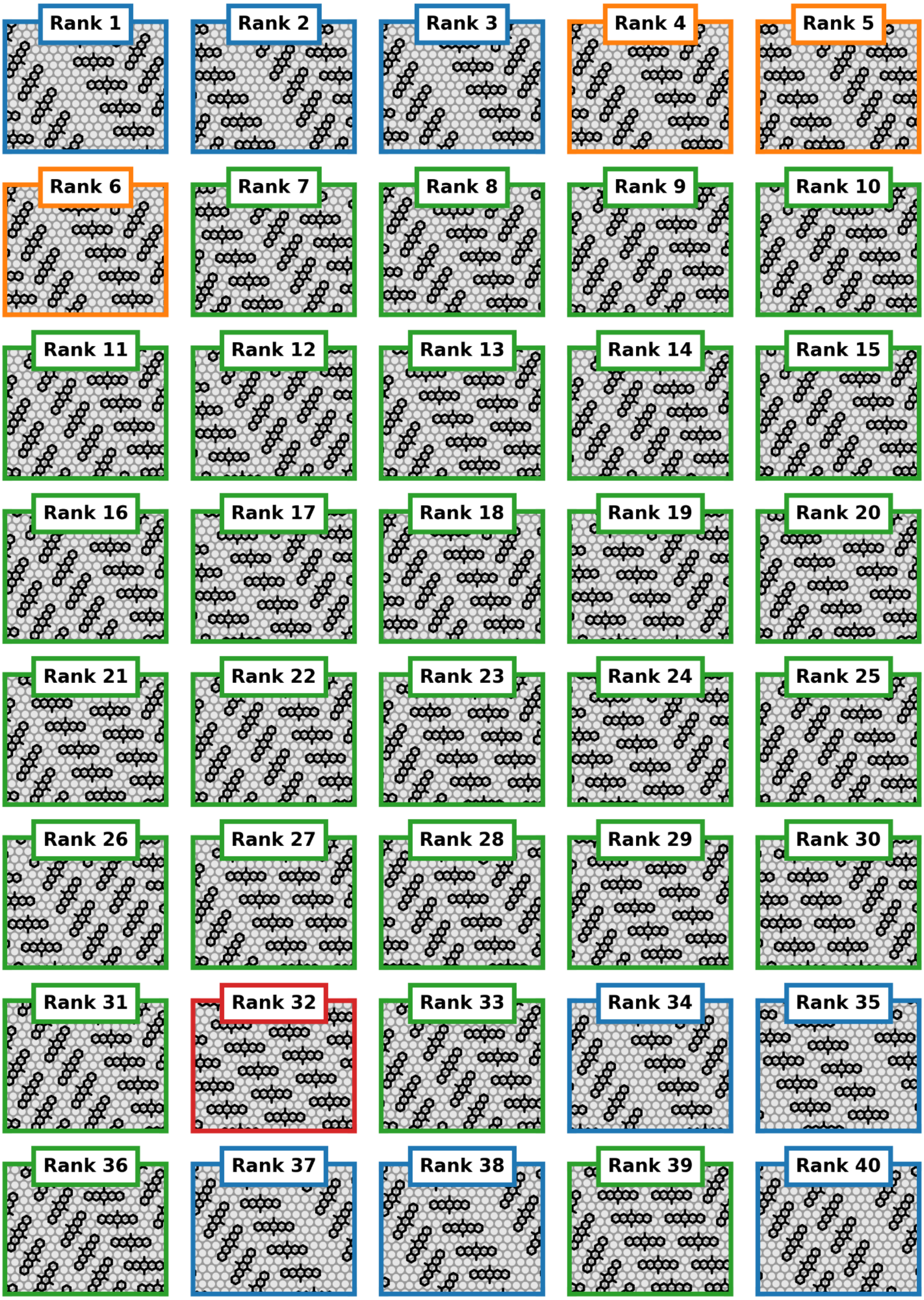

Figure S7: Visualization of all 40 best motifs for P2O. The colors are the same as for Figure 5: Blue corresponds to loosely packed motifs (146.9 Å^2/molecule), orange to slightly tighter packed motifs (136.4 Å^2/molecule), green are densely packed motifs (125.9 Å^2/molecule) and red is the one we compare to experiment because it is the lowest ranked structure without kinks.

4 Unit Cell Comparison

Table 1 shows the most important unit cells presented in Figure 4, a thorough analysis of all presented unit cells and uncertainties of the epitaxy matrices is given in Table S2. A graphical representation of the nomenclature of the unit cell parameters used for Table 1 and Table S2 is presented in Figure S8. For the calculation of the lattice vector lengths in Table S2 we used the converged lattice constant of our calculations, which amounts to 4.019 Å for the conventional unit cell (corresponding to a minimal Ag-Ag distance of 2.842 Å).

The experimental uncertainties of the matrix elements indicate the simple standard deviations of the values, while each value represents the result of a directly optimized parameter of the fit routine used. Only the matrix elements for A2O are derived by the combination of two different matrices (as shown in Figure S5). Therefor the uncertainties of this matrix are obtained *via* Gaussian error propagation as well as the uncertainties of the lattice parameters.

Table S2: Detailed comparison of experimental (grey background) and theoretical unit cells.

	a_1 [Å]	a_2 [Å]	Γ [°]	θ [°]	A [Å²]	Epitaxy
B2O I small	6.875(5)	6.603(3)	119.88(5)	-14.28(2)	39.36(4)	$\begin{pmatrix} 2.000(1) & -0.689(1) \\ 0.667(1) & 2.584(1) \end{pmatrix}$
	6.512	6.512	98.21	10.89	41.97	$\begin{pmatrix} 2.5 & 0.5 \\ 0.5 & 2.5 \end{pmatrix}$
B2O I large (Motif I)	11.926(8)	13.21(1)	91.21(8)	14.40(5)	157.5(2)	$\begin{pmatrix} 4.667(2) & 1.205(3) \\ 1.334(2) & 5.169(3) \end{pmatrix}$
	13.024	13.024	98.21	10.89	167.87	$\begin{pmatrix} 5 & 1 \\ 1 & 5 \end{pmatrix}$
B2O II small	6.875(5)	6.603(3)	119.88(5)	-14.28(2)	39.36(4)	$\begin{pmatrix} 2.000(1) & -0.689(1) \\ 0.667(1) & 2.584(1) \end{pmatrix}$
	6.512	7.211	121.07	-10.89	40.22	$\begin{pmatrix} 2 & -0.5 \\ 0.5 & 2.75 \end{pmatrix}$
B2O II large (Motif II)	13.75(1)	11.448(6)	88.51(7)	-14.30(3)	157.4(2)	$\begin{pmatrix} 4.000(3) & -1.380(2) \\ 3.334(2) & 4.476(2) \end{pmatrix}$
	13.024	12.388	94.31	-10.89	160.88	$\begin{pmatrix} 4 & -1 \\ 3 & 5 \end{pmatrix}$
A2O	24.6(3)	24.9(2)	120(1)	-36.5(4)	530(10)	$\begin{pmatrix} 3.99(6) & -5.94(6) \\ 5.98(8) & 10.04(8) \end{pmatrix}$
	24.776	24.776	120.00	-36.57	531.60	$\begin{pmatrix} 4 & -6 \\ 6 & 10 \end{pmatrix}$
P2O	14.96(2)	8.156(4)	95.92(8)	40.78(3)	121.3(2)	$\begin{pmatrix} 2.000(1) & -3.969(6) \\ 3.000(1) & 2.719(2) \end{pmatrix}$
	15.038	8.526	100.89	40.89	125.90	$\begin{pmatrix} 2 & -4 \\ 3 & 3 \end{pmatrix}$

a1, a2: Lengths of lattice vectors derived from the epitaxy matrices in combination with the theoretical substrate lattice vectors; Γ: angle of the adsorbate unit cell; θ: angle between a1 and the primitive substrate axis. Experimental uncertainties are indicated with parentheses.

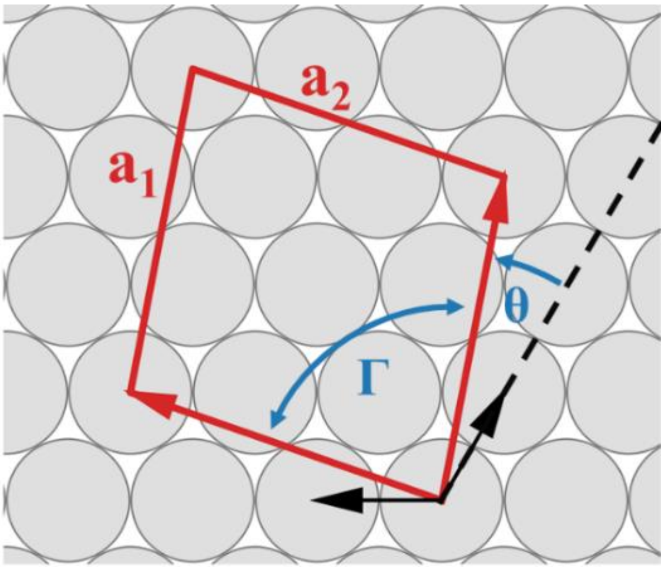

Figure S8: Visual explanation of the unit cell parameters used for cell comparison. a_1 and a_2 are the lengths of the unit cell lattice; Γ is defined as the enclosing angle of the unit cell vectors; θ represents the angle between the first lattice vector and a primitive substrate axis, negative values correspond to angles in anticlockwise direction.

5 Additional Pair Interaction Information

Figure 3 already visualized interactions of pairs of molecules with parallel alignment. In Figure S9 a selection of interaction maps for pairs with non-equal orientation is presented. To get an idea of the relationship between relative molecular orientation and interaction energies, in Figure S10 we visualize all intermolecular interaction energies for all three different molecules with respect to the relative orientation between two molecules within a pair.

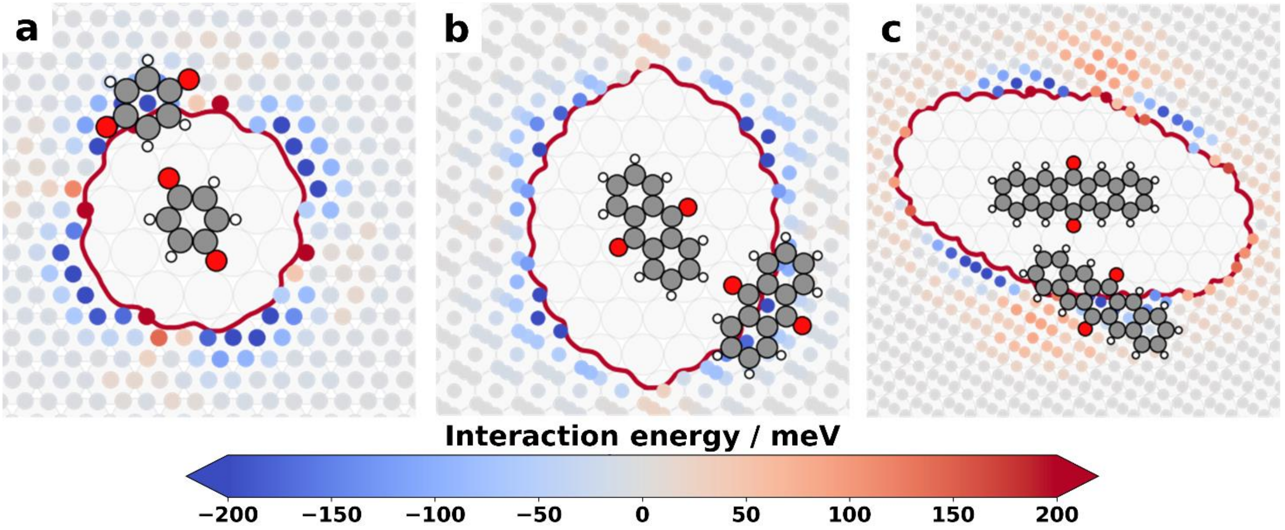

Figure S9: Total pairwise interaction energies for non-equivalent molecular orientation. Each circle represents a possible pairwise interaction between the central molecule and an adjacent molecule centered at the circle position for (a) B2O, (b) A2O and (c) P2O. The red contour shows the minimal distance before a pair is considered colliding. The circle color indicates the corresponding interaction energy.

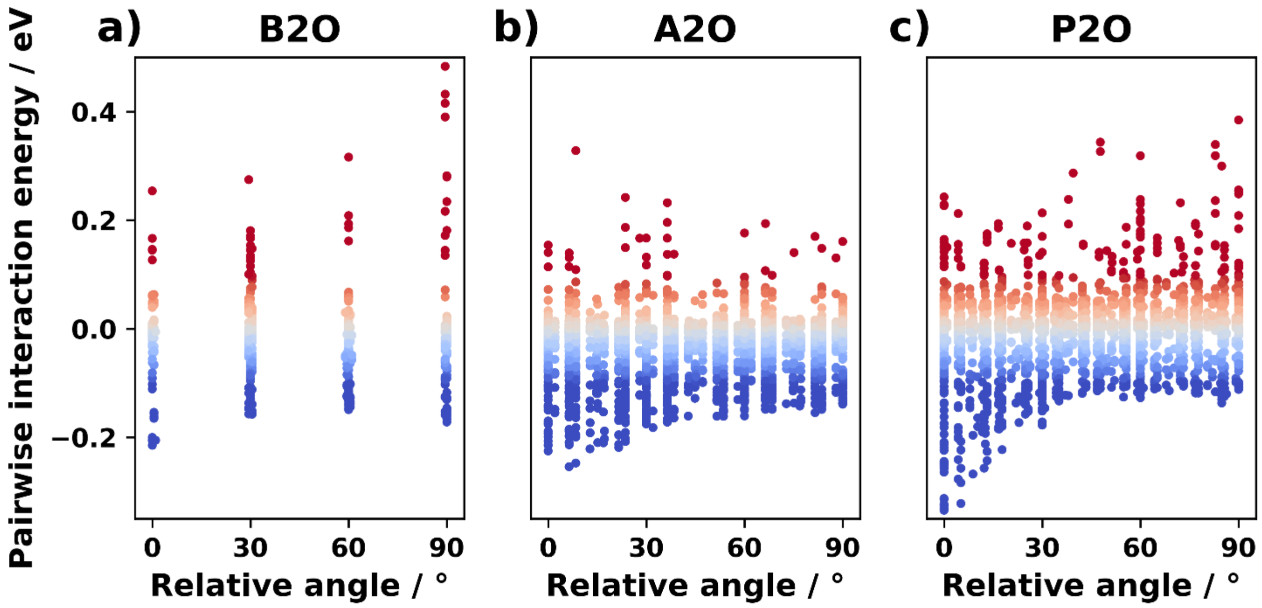

Figure S10: Visualization of all pairwise interaction energies for all three molecules dependent on the relative orientation between the pair. Each point indicates a unique pairwise interaction. The coloring indicates the energetic beneficiality of each pair and serves solely as a guide to the eye. Due to the D_{2h} symmetry of the individual molecules, all angles could be mapped into a range of 0 to 90 degree.

6 Symmetry Considerations of the Predicted Structures

As another interesting side note, for the three molecules, the best motifs also belong to different surface symmetry groups as can be seen in Figure S11. Here, A2O is the only molecule to exhibit an energetically beneficial structure with threefold symmetry. For P2O we do not find a threefold symmetric structure in the set of possible motifs. The reason for this is that intractably large unit cells with low coverage would be necessary to build such structures. As we could also not observe any threefold-symmetric structures in our experiments and the difference in intermolecular interaction energies between 0 and 60 degree rotation is far larger for P2O compared to A2O (see Figure S10b and c), we do not expect (tight packed) hexagonal structures to be energetically favorable for P2O. The motif for P2O which we use for comparison with the experiment (see Figure 4) also exhibits a C_1 symmetry.

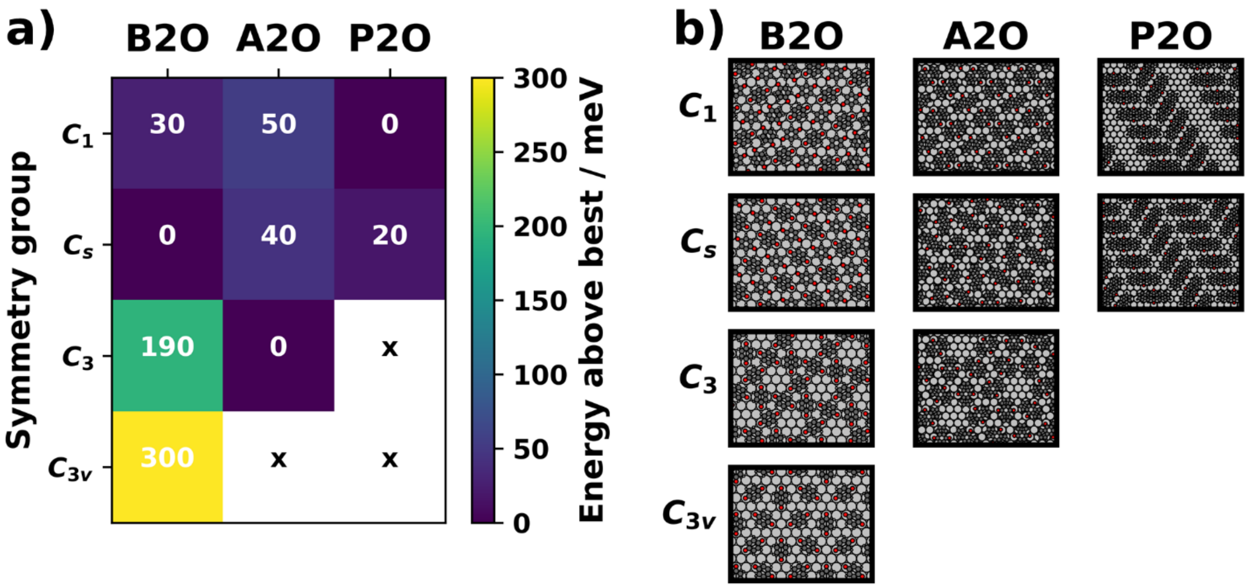

Figure S11: a) Best motif for each molecule for all different 2D symmetry groups available in the set of possible motifs. The symmetry groups are specified by their Schoenflies notation. The color and inset number represent the energetic difference (in meV) to the overall best motif found for each molecule; b) Graphical representation of the best motifs for each symmetry group shown in a).

References

(1) Blum, V.; Gehrke, R.; Hanke, F.; Havu, P.; Havu, V.; Ren, X.; Reuter, K.; Scheffler, M. *Ab Initio* Molecular Simulations with Numeric Atom-Centered Orbitals. *Comput. Phys. Commun.* **2009**, *180* (11), 2175–2196. https://doi.org/https://doi.org/10.1016/j.cpc.2009.06.022.

(2) Havu, V.; Blum, V.; Havu, P.; Scheffler, M. Efficient O(N) Integration for All-Electron Electronic Structure Calculation Using Numeric Basis Functions. *J. Comput. Phys.* **2009**, *228* (22), 8367–8379. https://doi.org/10.1016/J.JCP.2009.08.008.

(3) Marek, A.; Blum, V.; Johanni, R.; Havu, V.; Lang, B.; Auckenthaler, T.; Heinecke, A.; Bungartz, H.-J.; Lederer, H. The ELPA Library: Scalable Parallel Eigenvalue Solutions for Electronic Structure Theory and Computational Science. *J. Phys. Condens. Matter* **2014**, *26* (21), 213201. https://doi.org/10.1088/0953-8984/26/21/213201.

(4) Ihrig, A. C.; Wieferink, J.; Zhang, I. Y.; Ropo, M.; Ren, X.; Rinke, P.; Scheffler, M.; Blum, V. Accurate Localized Resolution of Identity Approach for Linear-Scaling Hybrid Density Functionals and for Many-Body Perturbation Theory. *New J. Phys.* **2015**, *17* (9), 093020. https://doi.org/10.1088/1367-2630/17/9/093020.

(5) Yu, V. W.; Corsetti, F.; García, A.; Huhn, W. P.; Jacquelin, M.; Jia, W.; Lange, B.; Lin, L.; Lu, J.; Mi, W.; Seifitokaldani, A.; Vázquez-Mayagoitia, Á.; Yang, C.; Yang, H.; Blum, V. ELSI: A Unified Software Interface for Kohn–Sham Electronic Structure Solvers. *Comput. Phys. Commun.* **2018**, *222*, 267–285. https://doi.org/10.1016/J.CPC.2017.09.007.

(6) Todorović, M.; Gutmann, M. U.; Corander, J.; Rinke, P. Bayesian Inference of Atomistic Structure in Functional Materials. *npj Comput. Mater.* **2019**, *5* (1), 35. https://doi.org/10.1038/s41524-019-0175-2.

(7) Heimel, G.; Duhm, S.; Salzmann, I.; Gerlach, A.; Strozecka, A.; Niederhausen, J.; Bürker, C.; Hosokai, T.; Fernandez-Torrente, I.; Schulze, G.; Winkler, S.; Wilke, A.; Schlesinger, R.; Frisch, J.; Bröker, B.; Vollmer, A.; Detlefs, B.; Pflaum, J.; Kera, S.; Franke, K.J.; *et al.* Charged and Metallic Molecular Monolayers through Surface-Induced Aromatic Stabilization. *Nat. Chem.* **2013**, *5* (3), 187–194. https://doi.org/10.1038/nchem.1572.

(8) Hörmann, L.; Jeindl, A.; Egger, A. T.; Scherbela, M.; Hofmann, O. T. SAMPLE: Surface Structure Search Enabled by Coarse Graining and Statistical Learning. *Comput. Phys. Commun.* **2019**. https://doi.org/https://doi.org/10.1016/j.cpc.2019.06.010.

(9) Gruenewald, M.; Peuker, J.; Meissner, M.; Sojka, F.; Forker, R.; Fritz, T. Impact of a Molecular Wetting Layer on the Structural and Optical Properties of Tin(II)-Phthalocyanine Multilayers on Ag(111). *Phys. Rev. B* **2016**, *93* (11), 115418. https://doi.org/10.1103/PhysRevB.93.115418.

(10) Sojka, F.; Meissner, M.; Zwick, C.; Forker, R.; Fritz, T. Determination and Correction of Distortions and Systematic Errors in Low-Energy Electron Diffraction. *Rev. Sci. Instrum.* **2013**, *84* (1), 015111. https://doi.org/10.1063/1.4774110.
